# Supplementary material for: Characterizing open-ended evolution through undecidability mechanisms in random Boolean networks
Source: NPJ Syst Biol Appl. 2026 Jun 23;12:152. doi: 10.1038/s41540-026-00770-8 (PMC13654937; doi:10.1038/s41540-026-00770-8)
Supplement: Supplementary file 1 [file 41540_2026_770_MOESM1_ESM.pdf]

Supplementary Material

Characterizing Open-Ended Evolution Through Undecidability Mechanisms in Random Boolean Networks

Amahury J. López-Díaz[1,*], Pedro Juan Rivera Torres[2, 4], Gerardo Febres[1,3], and Carlos Gershenson[1]

[1] *School of Systems Science and Industrial Engineering, Binghamton University, 4400 Vestal Pkwy E, Binghamton, NY 13902, USA*
[2] *Department of Computer Science and Automation, Universidad de Salamanca, 1 Patio de Escuelas, Salamanca, 37008, Spain*
[3] *Department of Processes and Systems, Universidad Simón Bolívar, C479+36J, Caracas, Miranda, Venezuela*
[4] *St. Edmund's College, University of Cambridge, Cambridge, CB3 0BN, United Kingdom*
[*] *Corresponding Author*
alpez@binghamton.edu

June 7, 2026

The present document includes experiments we have carried out for the paper "Characterizing Open-Ended Evolution Through Undecidability Mechanisms in Random Boolean Networks," where we explore the behavior of open-endedness using multiple mechanisms based on non-classical logics.

Abstract

Discrete dynamical models underpin systems biology, but we still lack substrate-agnostic diagnostics for identifying finite-horizon dynamical signatures that may be relevant to open-ended evolution (OEE), such as the recurrent production of novel phenotypic states rather than rapid settling or unstructured noise. We introduce a simple, model-independent metric, Ω, that summarizes the residence-time-weighted contribution of attractor cycle lengths across the sequence of recurrent episodes realized within a finite observation window. Ω is zero for single-attractor dynamics and also vanishes for pure novelty without recurrence, while increasing when trajectories repeatedly enter multiple persistent cyclic phenotypes. Using Random Boolean Networks (RBNs) as a controlled testbed, we compare classical Boolean dynamics with biologically motivated non-classical mechanisms (probabilistic context switching, annealed rule mutation, paraconsistent logic, modal necessary/possible gating, and quantum-inspired superposition/paired-state coupling) under homogeneous and heterogeneous updating schemes. Our results support the view that undecidability-adjacent, state-dependent mechanisms—implemented as probabilistic context switching, modal necessity/possibility gating, paraconsistent logic, or quantum-inspired correlated branching—are enabling conditions for sustained novelty. At the end of our manuscript we outline a practical extension of Ω to continuous/hybrid state spaces, positioning Ω as a portable proxy for OEE in biological modeling and a guide for engineering evolvable synthetic circuits.

Shared experimental backbone (see Methods and Results in main text): unless stated otherwise, each point averages 1,000 independently sampled networks with $N = 100$ nodes, a random initial condition, mean connectivity $K \in [1.1, 4.5]$ in steps of 0.2, and horizon $T = 10^6$ time steps. The open-endedness metric is $\Omega = \frac{1}{T^2}\sum_{j=1}^{m} d_j k_j$, the residence-time-weighted sum of the cycle-lengths of each attractor A_j found within the window.

Horizon sensitivity (homogeneous and heterogeneous)

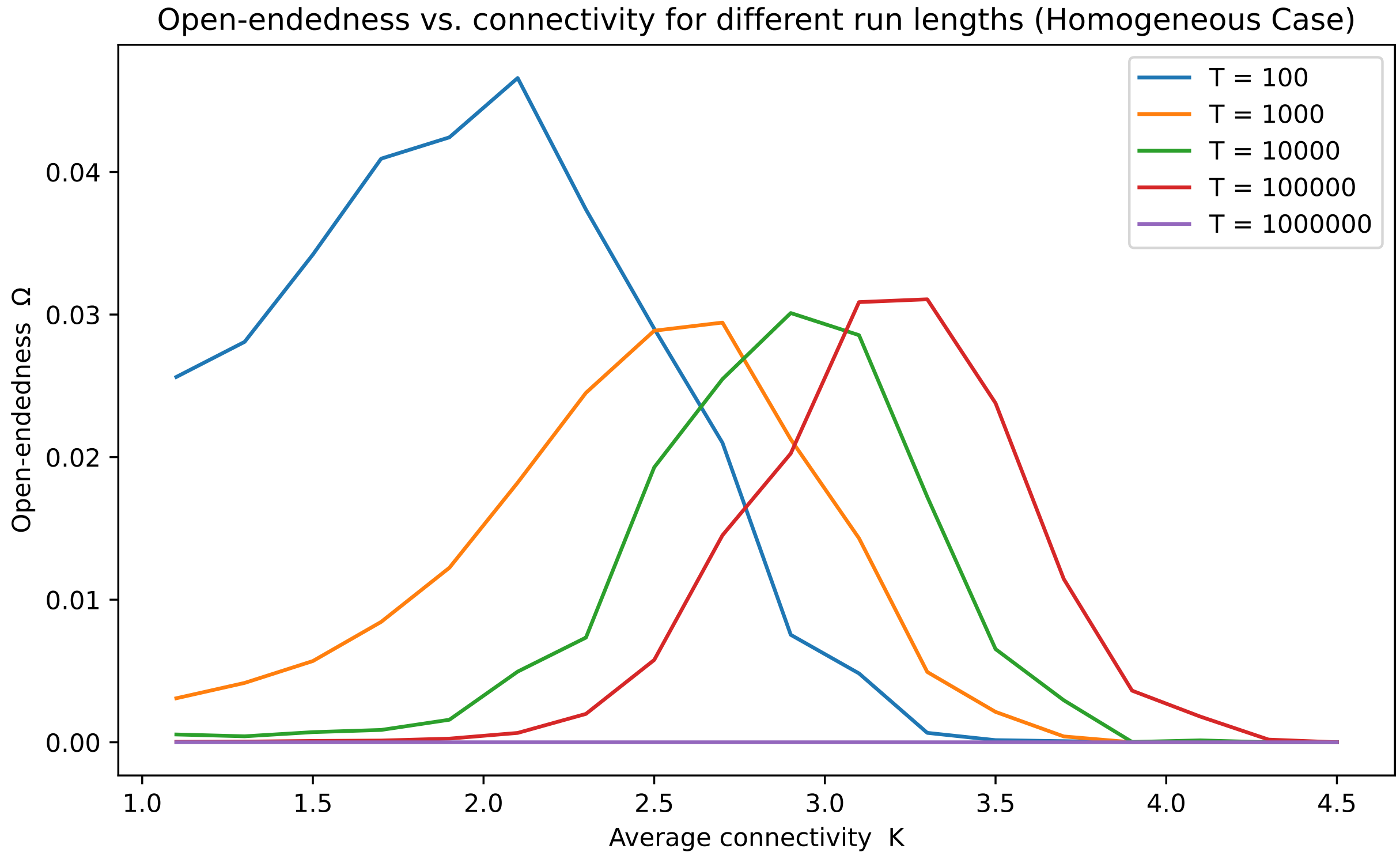

Supplementary Figure 1: **Horizon (T) sensitivity of $\Omega(K)$ under homogeneous settings.** Each curve shows the mean Ω across 1,000 networks at fixed K for different simulation lengths T (legend as plotted in the notebook). Curves largely overlap beyond the smallest T, indicating that $T = 10^6$ suffices to stabilize $\Omega(K)$ in this regime. Axes: x–axis is average connectivity K; y–axis is open-endedness Ω (dimensionless, residence-time-weighted cycle-length normalized by T^2).

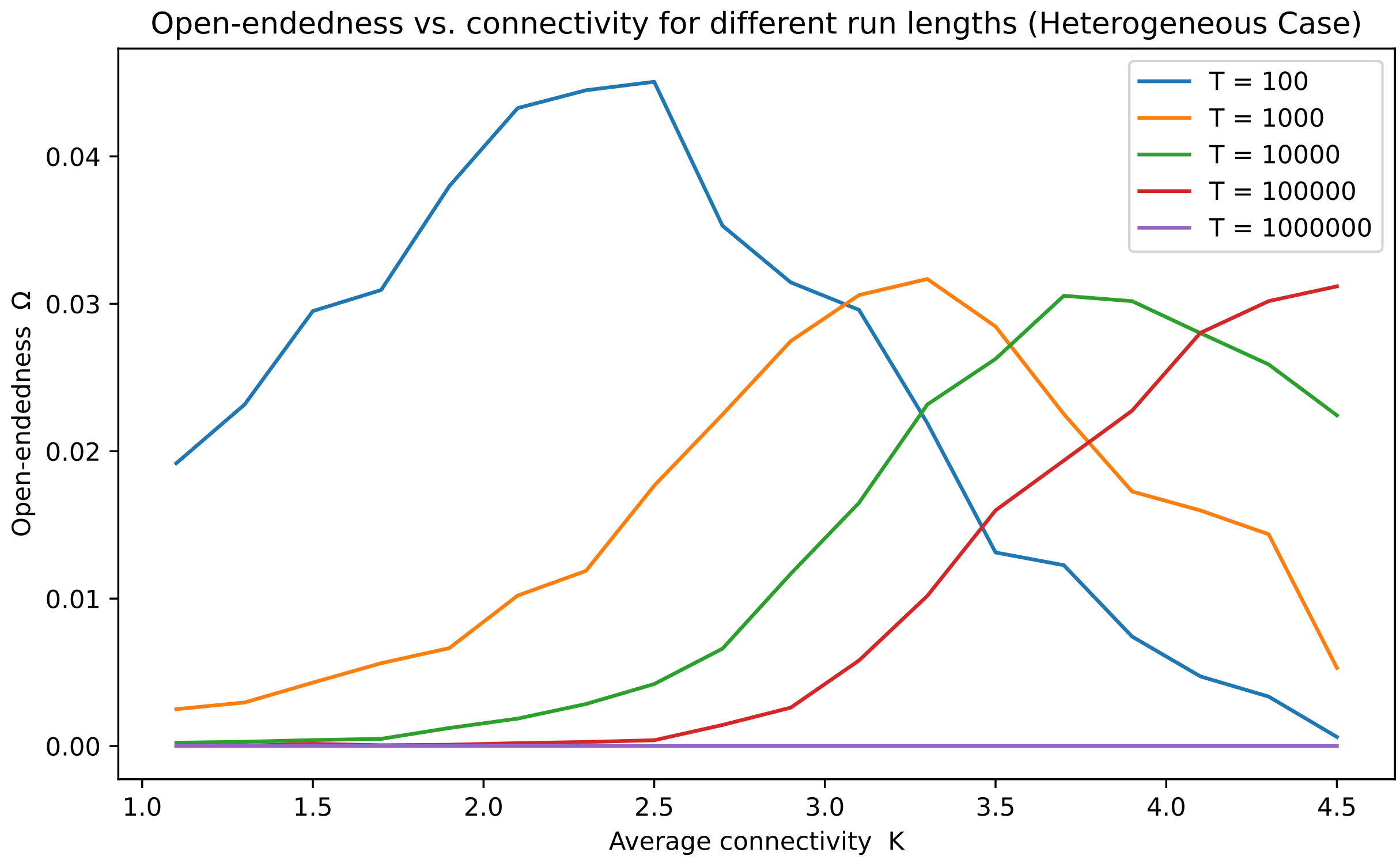

Supplementary Figure 2: **Horizon (T) sensitivity of $\Omega(K)$ under heterogeneous settings.** Same protocol as Fig. 1 but with Exponential in-degree and asynchronous updates. Ω remains near zero across K at long T when dynamics collapse to fixed points, justifying the large-T choice used elsewhere.

Probabilistic Boolean Networks (PBN): context count and switching probability

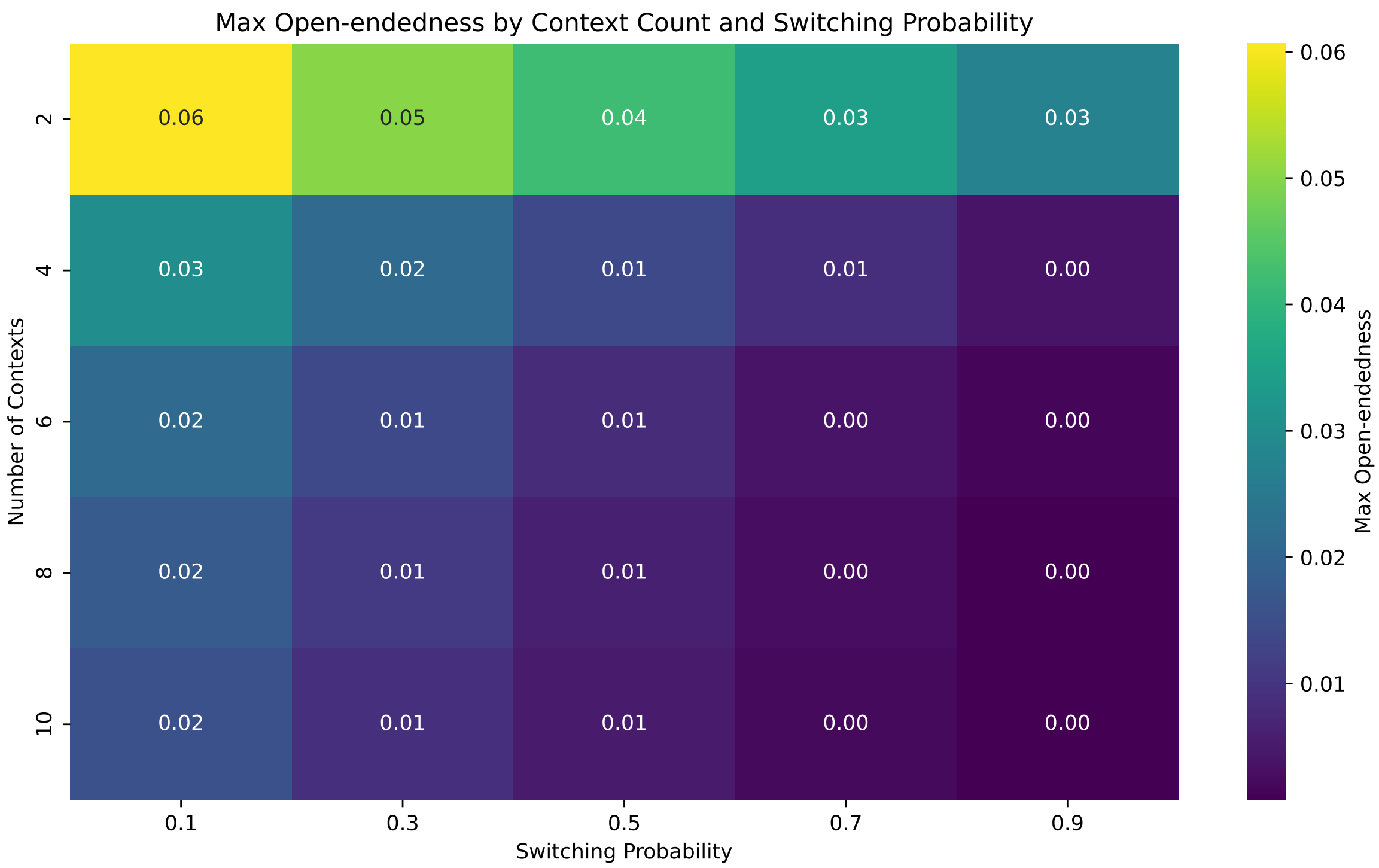

Supplementary Figure 3: **Maximum observed Ω over K as a function of PBN switching probability σ (x-axis) and number of deterministic contexts (y-axis).** Higher counts allow more distinct attractor families but excessive switching suppresses dwell times; a sweet spot emerges at low σ with few contexts. Color scale: maximum mean Ω attained across the K grid for each $(\sigma, \text{contexts})$.

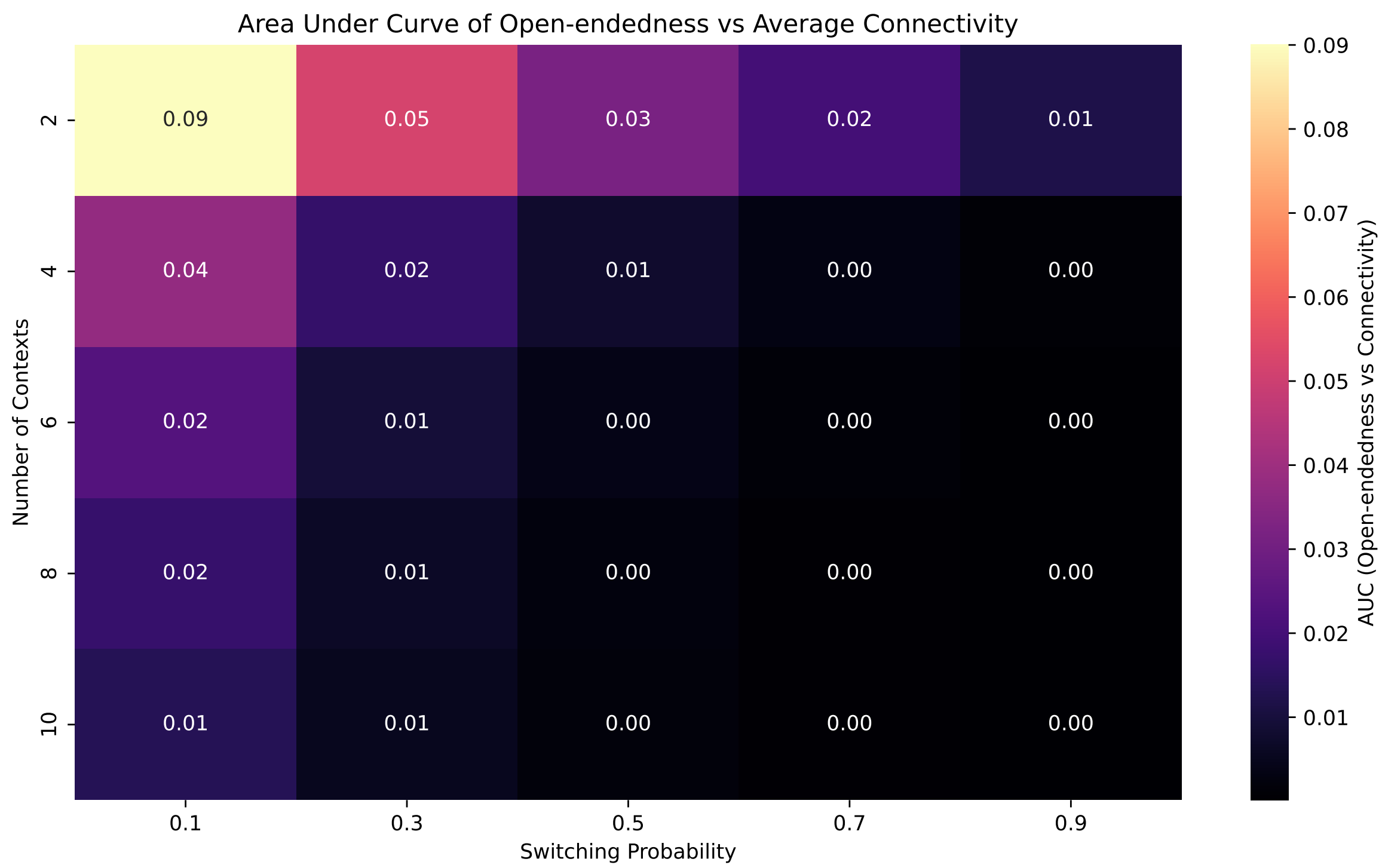

Supplementary Figure 4: **AUC of $\Omega(K)$ across K for each $(\sigma, \text{contexts})$. The area-under-curve condenses overall performance across connectivities. The optimal setting used in the main text is annotated in the notebook (typically contexts $= 2$, $\sigma = 0.1$ for the homogeneous case).**

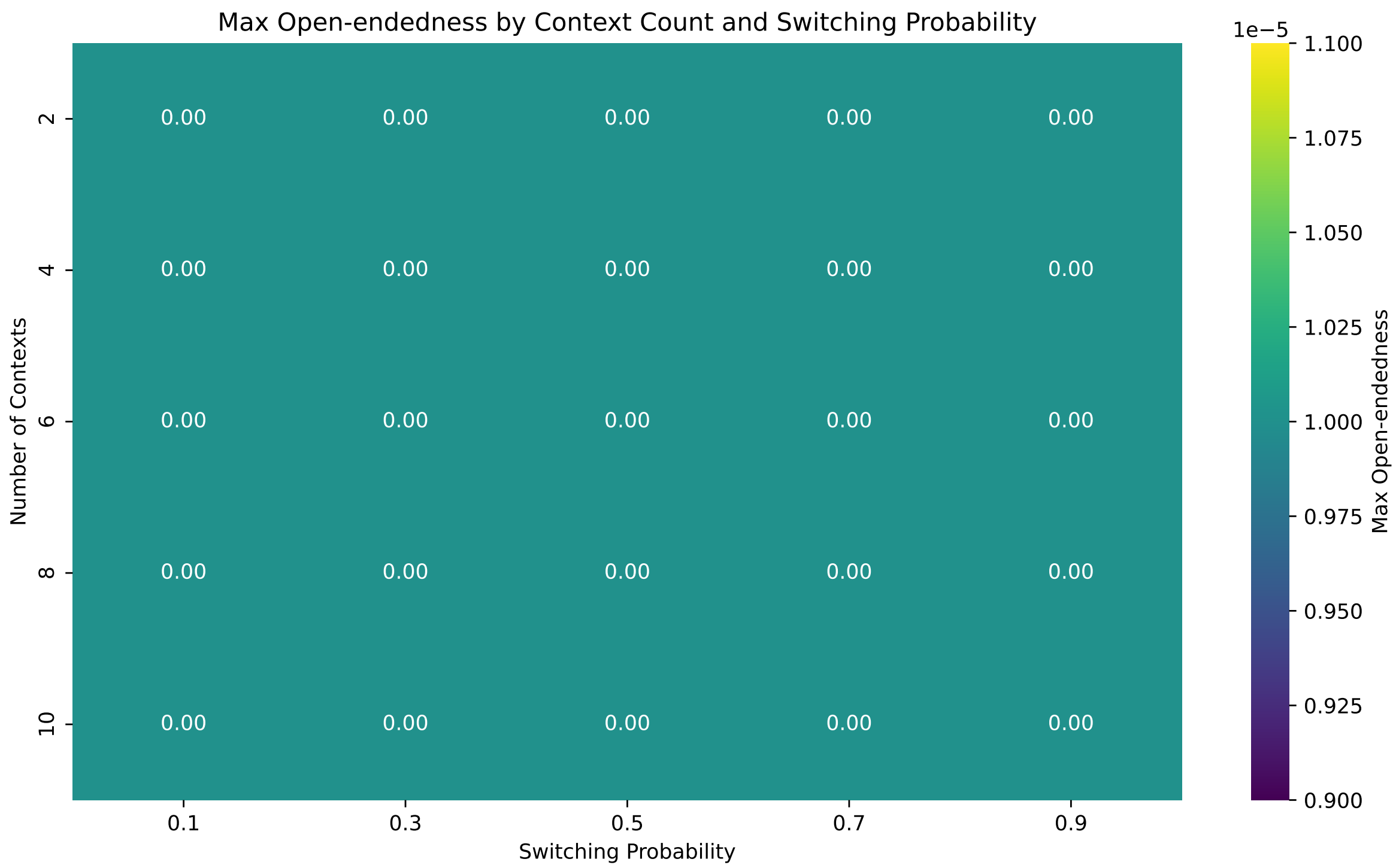

Supplementary Figure 5: **PBN (σ, contexts) landscape under heterogeneity.** Max-Ω remains low throughout, consistent with asynchronous dynamics favoring point attractors; switching cannot compensate if each context quickly collapses. Color scale and axes as in Fig. 3.

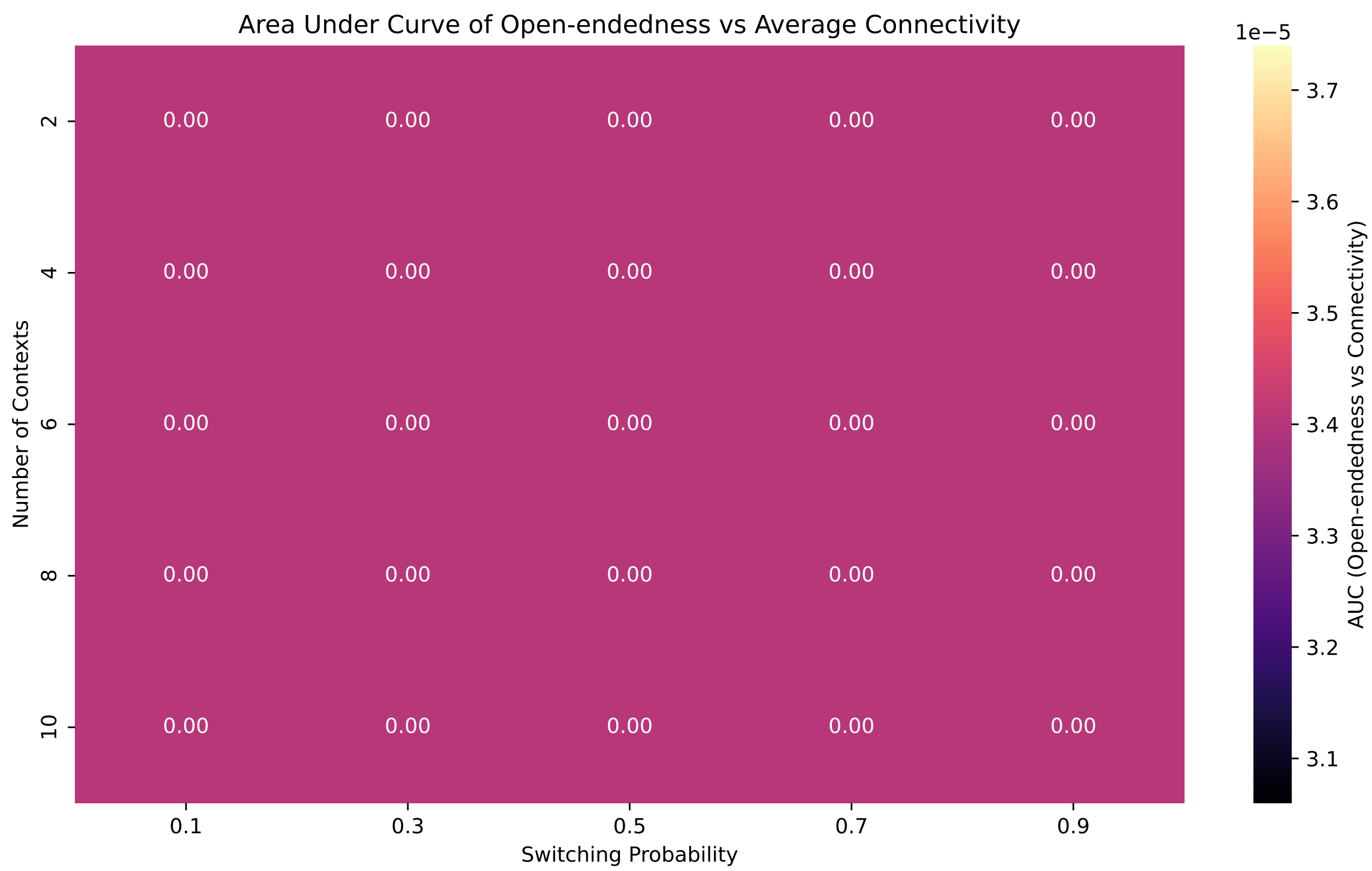

Supplementary Figure 6: **PBN AUC across (σ, contexts) under heterogeneity.** Confirms the collapse of Ω across K; the setting used in the main text (`SwitchProb=0.1_Contexts=2`) is reported for completeness.

Annealed Rule Mutation (ARM): mutation probability μ

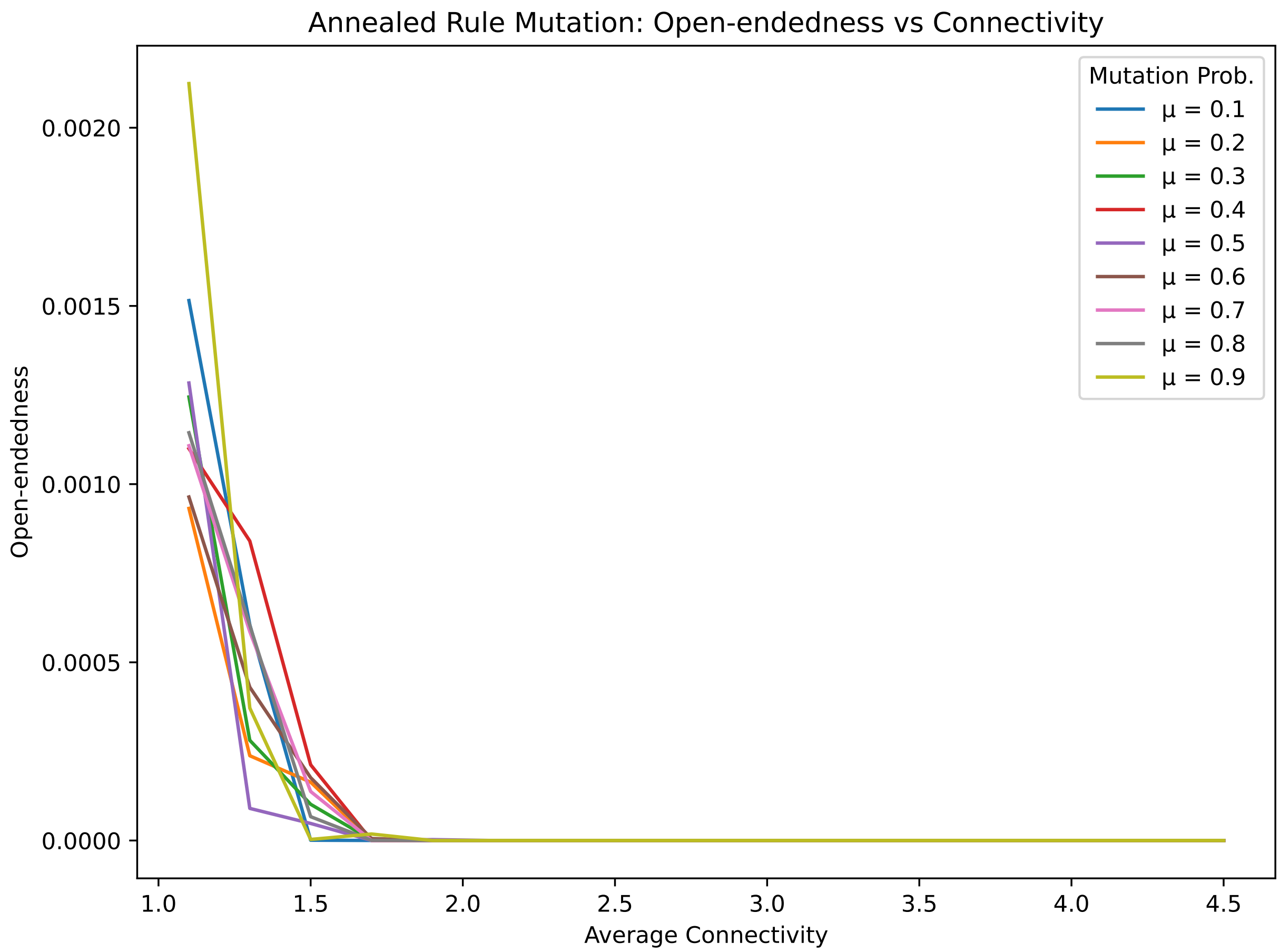

Supplementary Figure 7: $\Omega(K)$ **for several mutation probabilities** μ **(legend as plotted).** Increasing μ anneals away structured attractor geometry and reduces dwell-time×cycle-length, driving Ω down. Axes: K vs. Ω.

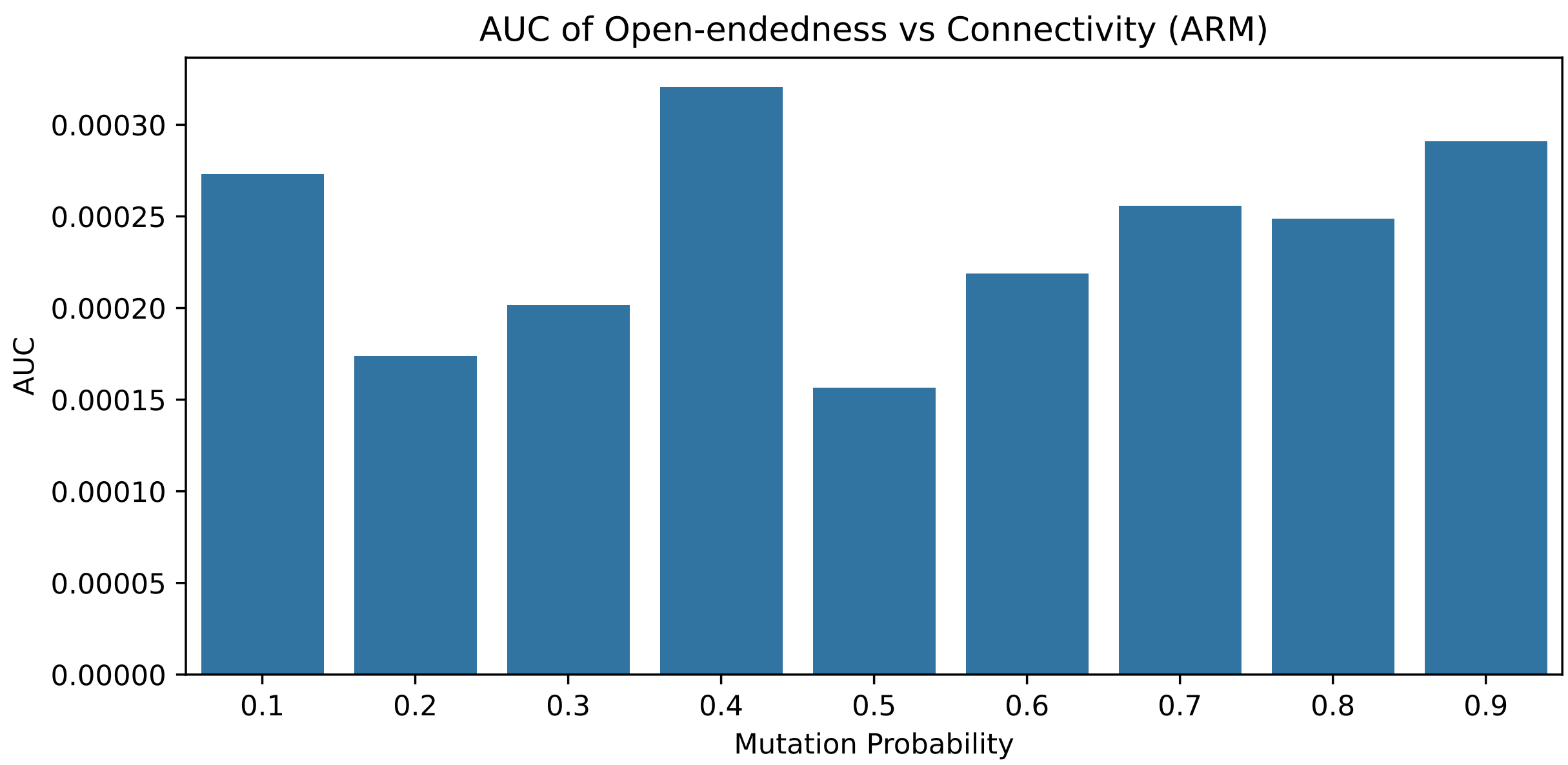

Supplementary Figure 8: **AUC of $\Omega(K)$ vs. μ.** The optimal (highest AUC) μ used in the main text is indicated in the notebook (homogeneous winner: `MutationProb=0.4`). Error bars omitted; each AUC aggregates the mean over K.

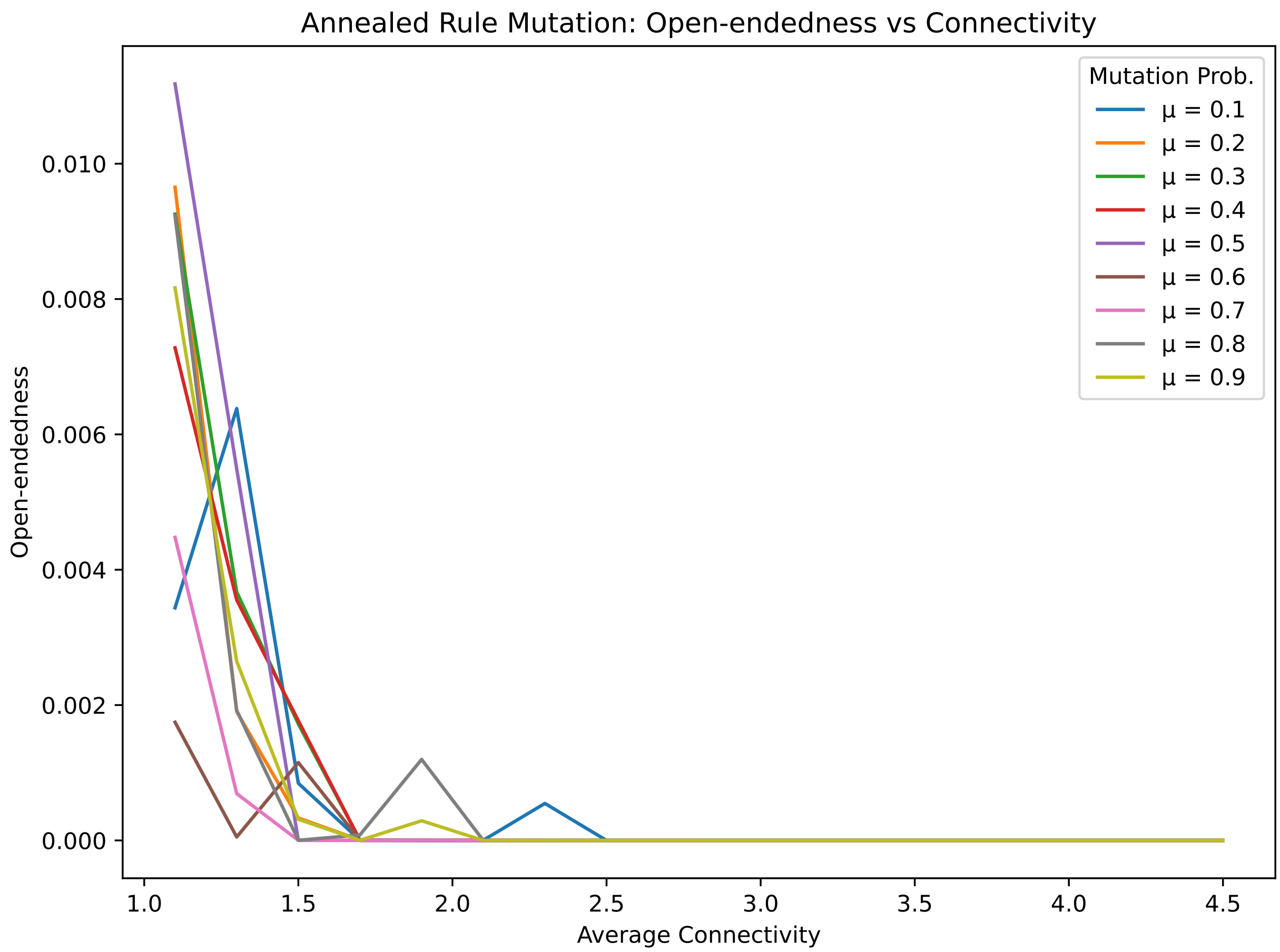

Supplementary Figure 9: $\Omega(K)$ **under ARM with heterogeneity.** Asynchrony accelerates convergence to fixed points; even moderate μ suppresses Ω.

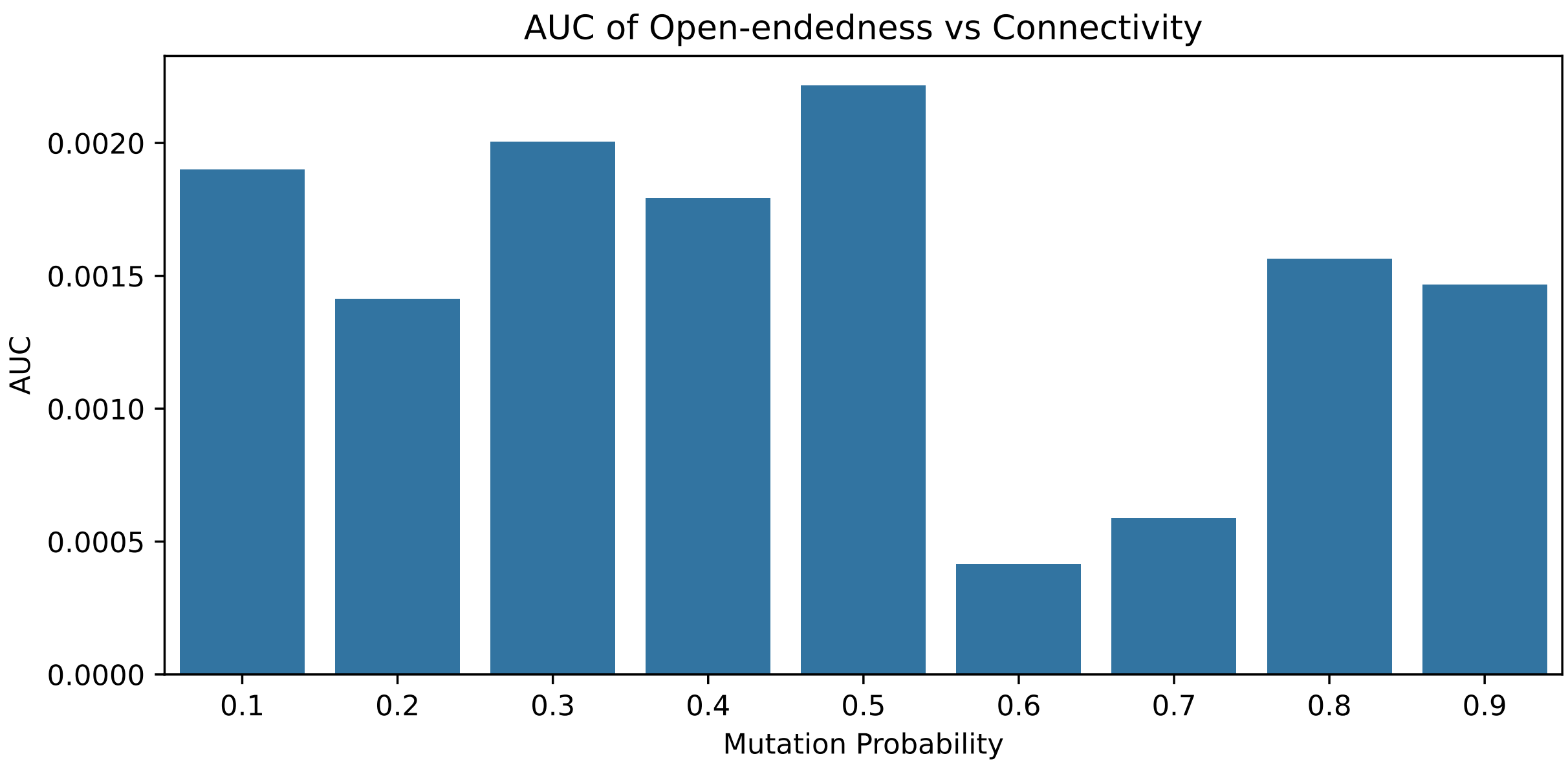

Supplementary Figure 10: **AUC vs. μ under heterogeneity.** The selected setting in the main text corresponds to `MutationProb=0.5`.

Quantum-inspired mechanism: superposition probability s_p and paired-state couplings e

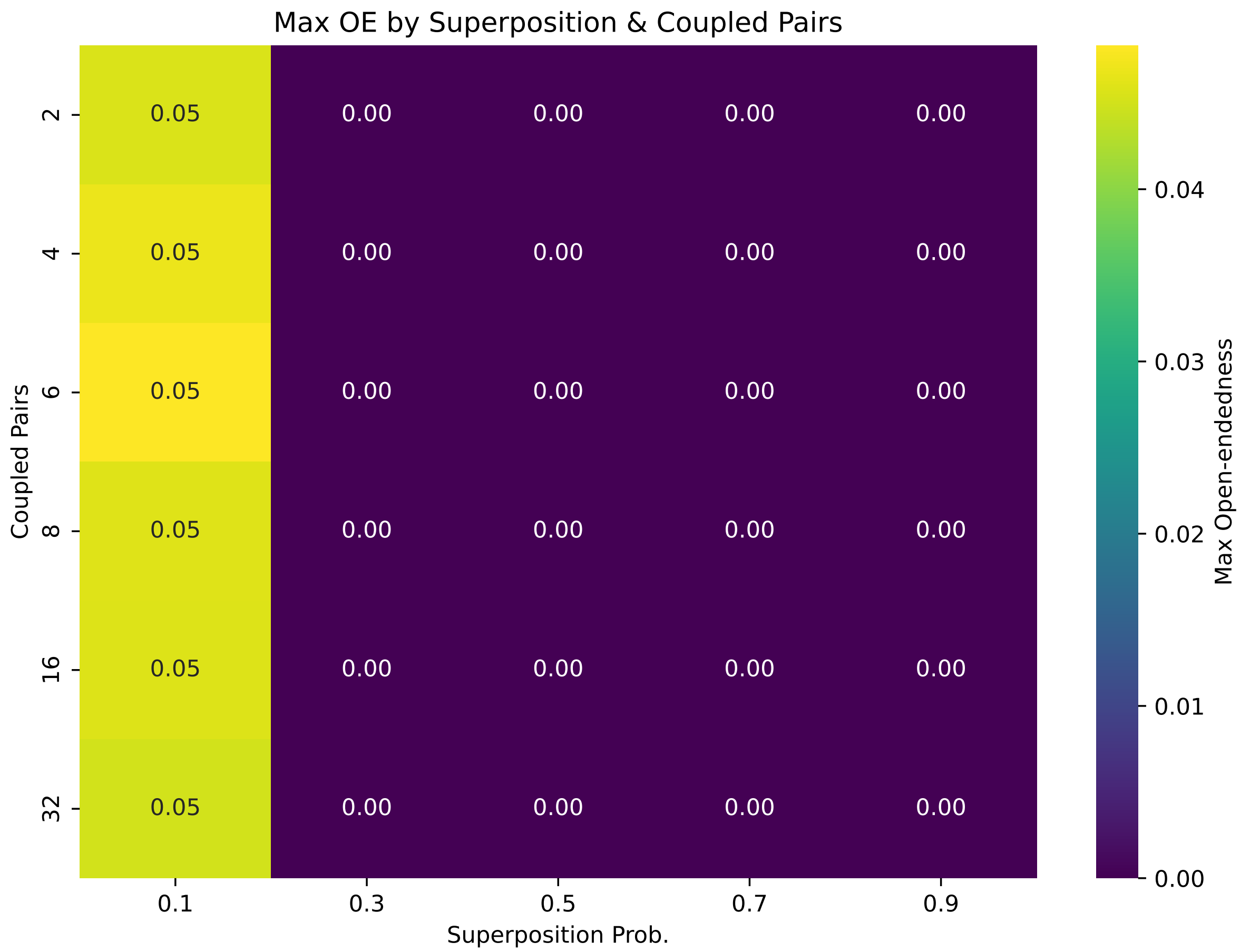

Supplementary Figure 11: **Maximum Ω across K for each (s_p, e) pair.** Small s_p with a few long-range pairs yields correlated branching without washing out structure; the notebook marks the peak (`Superposition=0.1_Paired=6`).

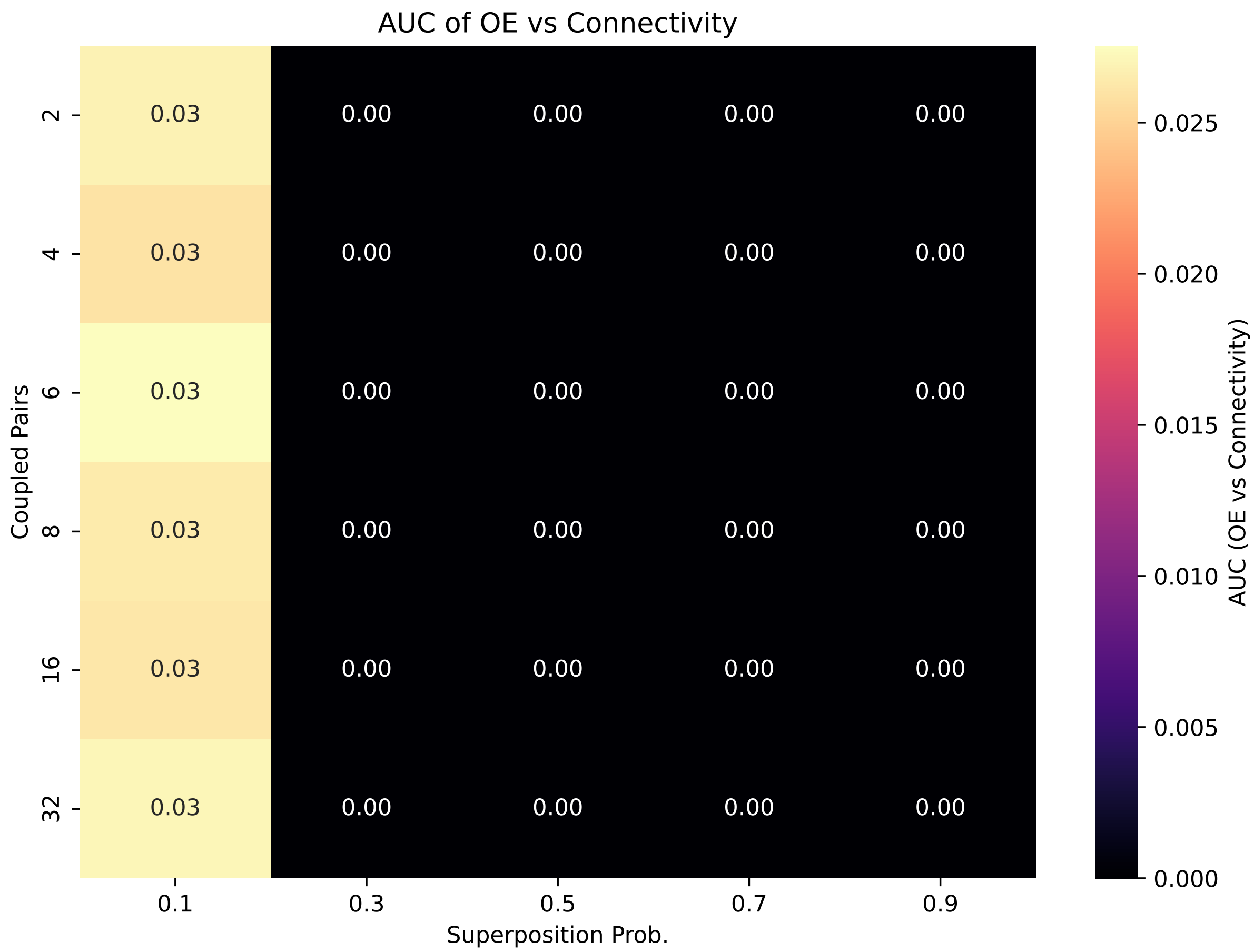

Supplementary Figure 12: **AUC across** (s_p, e)**.** Summarizes overall performance across connectivities for each parameter pair; the winning combination is consistent with Fig. 11.

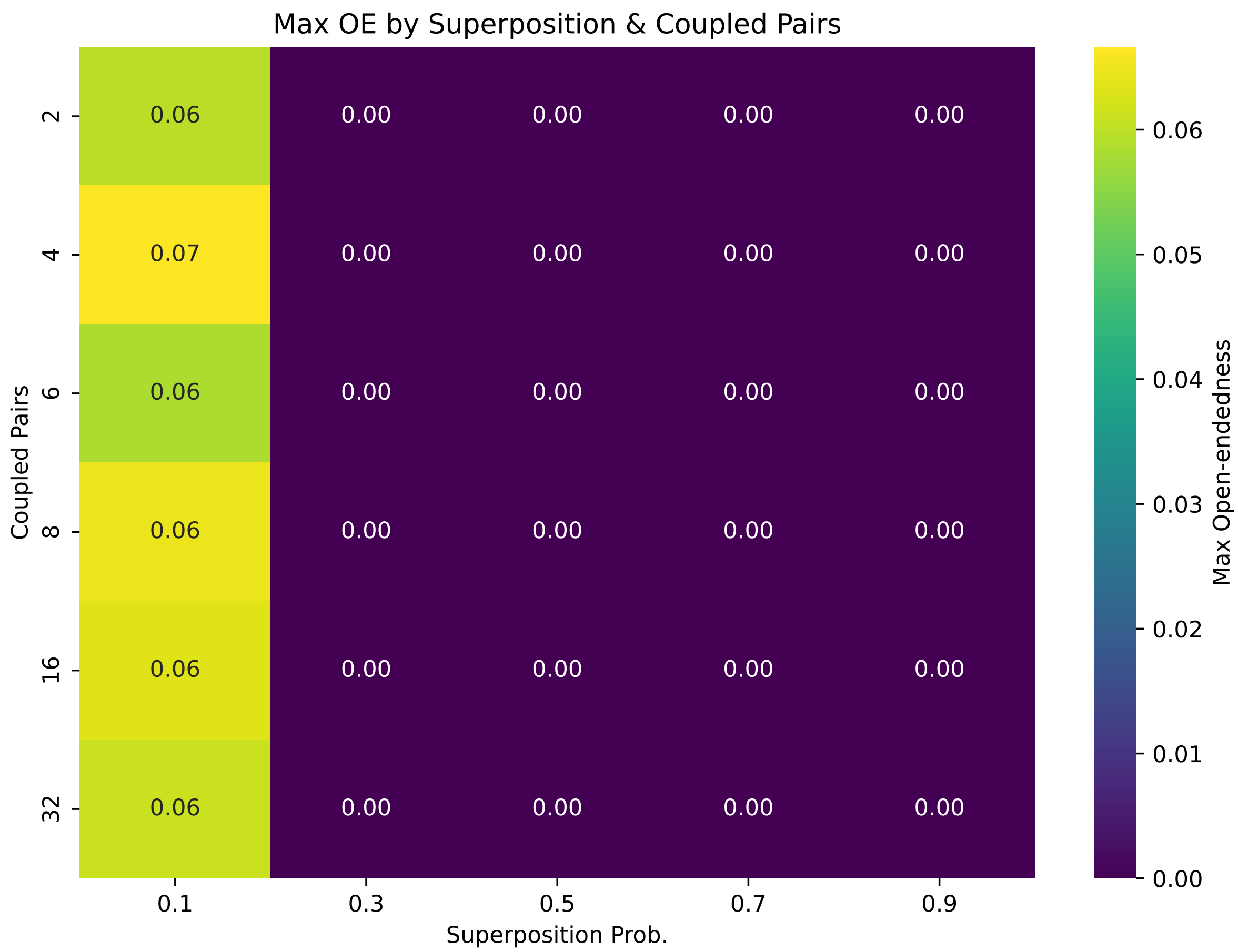

Supplementary Figure 13: **Max-Ω across** (s_p, e) **under heterogeneity.** Sparse wiring benefits more from correlated branching; the best combination used in the main text is annotated (`Superposition=0.1_Paired=32`).

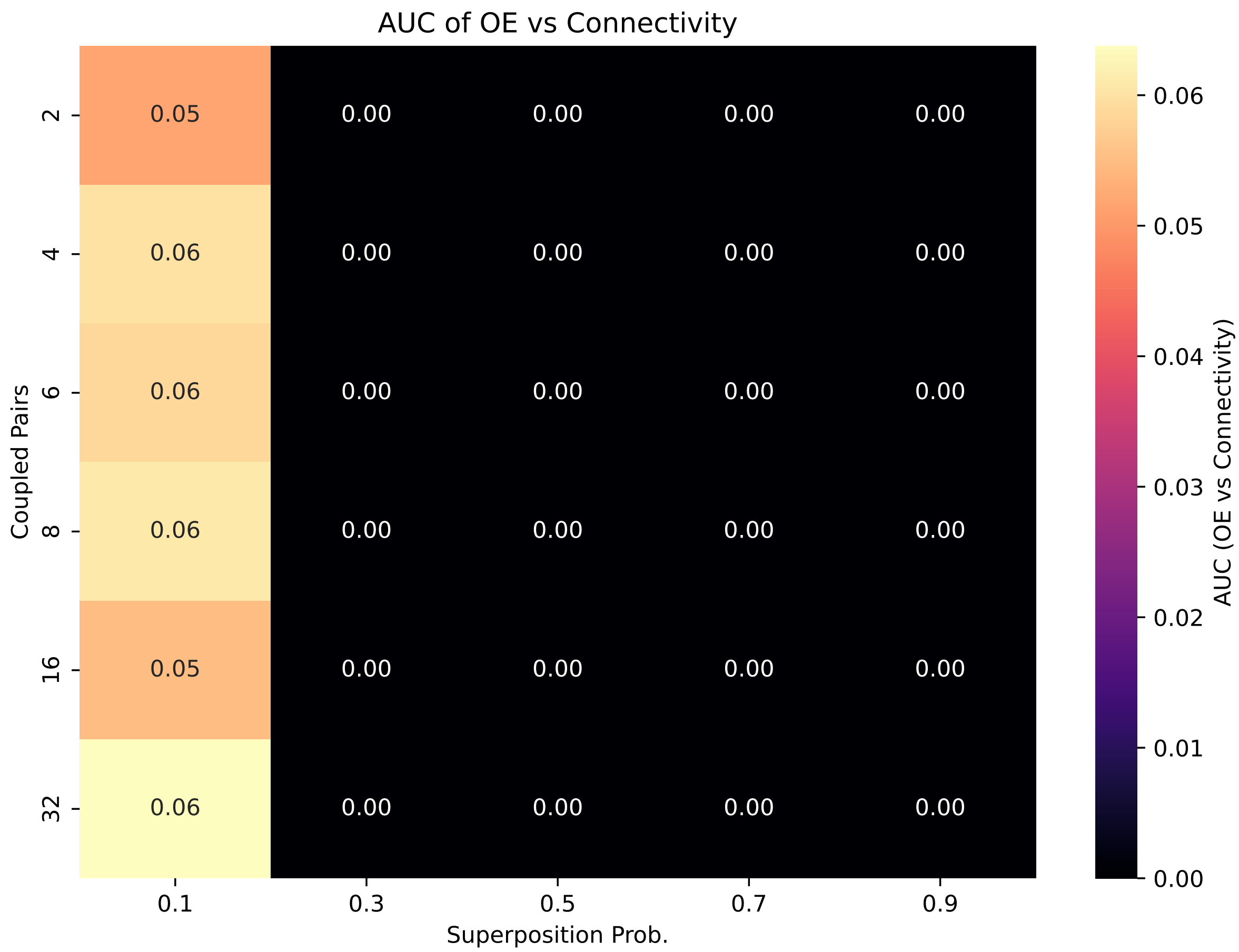

Supplementary Figure 14: **AUC across (s_p, e) under heterogeneity.** Confirms the peak at moderate s_p and larger e.

Paraconsistent logic: contradiction probability c

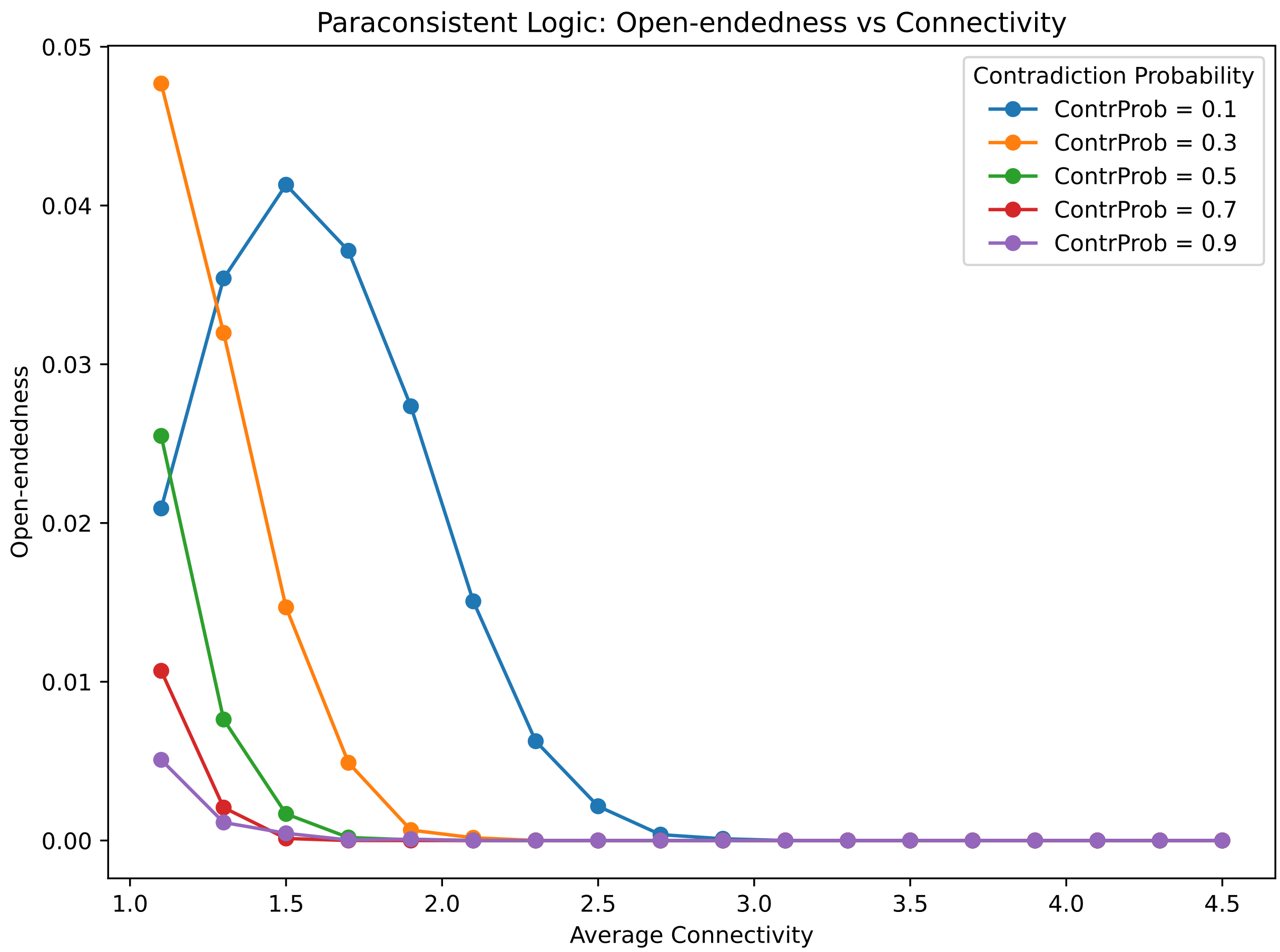

Supplementary Figure 15: **$\Omega(K)$ at several contradiction probabilities c.** Introducing inconsistency-tolerant tokens around the order-critical region ($K \approx 2$) sustains exploratory dynamics without runaway noise.

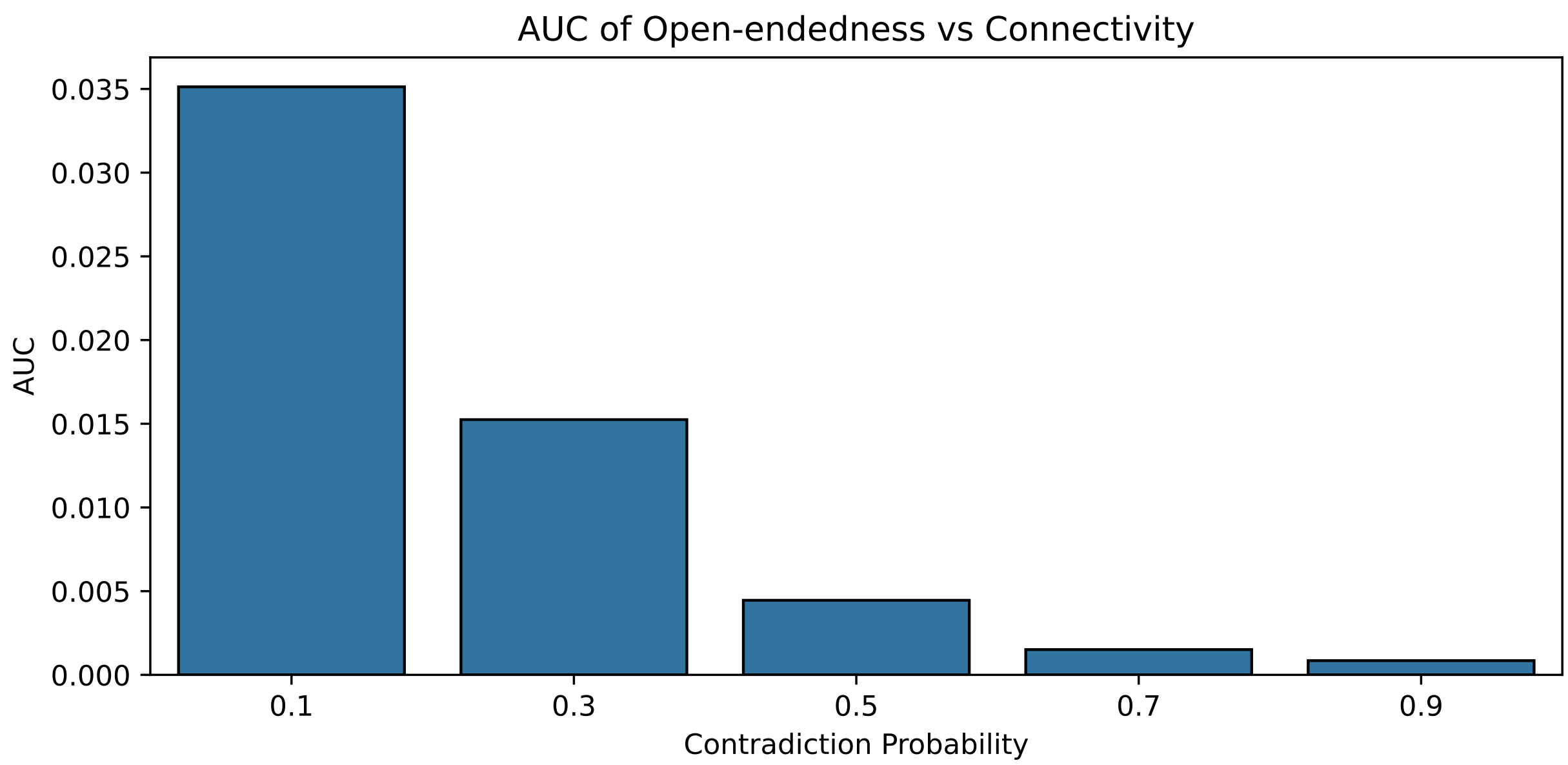

Supplementary Figure 16: **AUC vs. c.** The best value used in the main text is `ContrProb=0.1`.

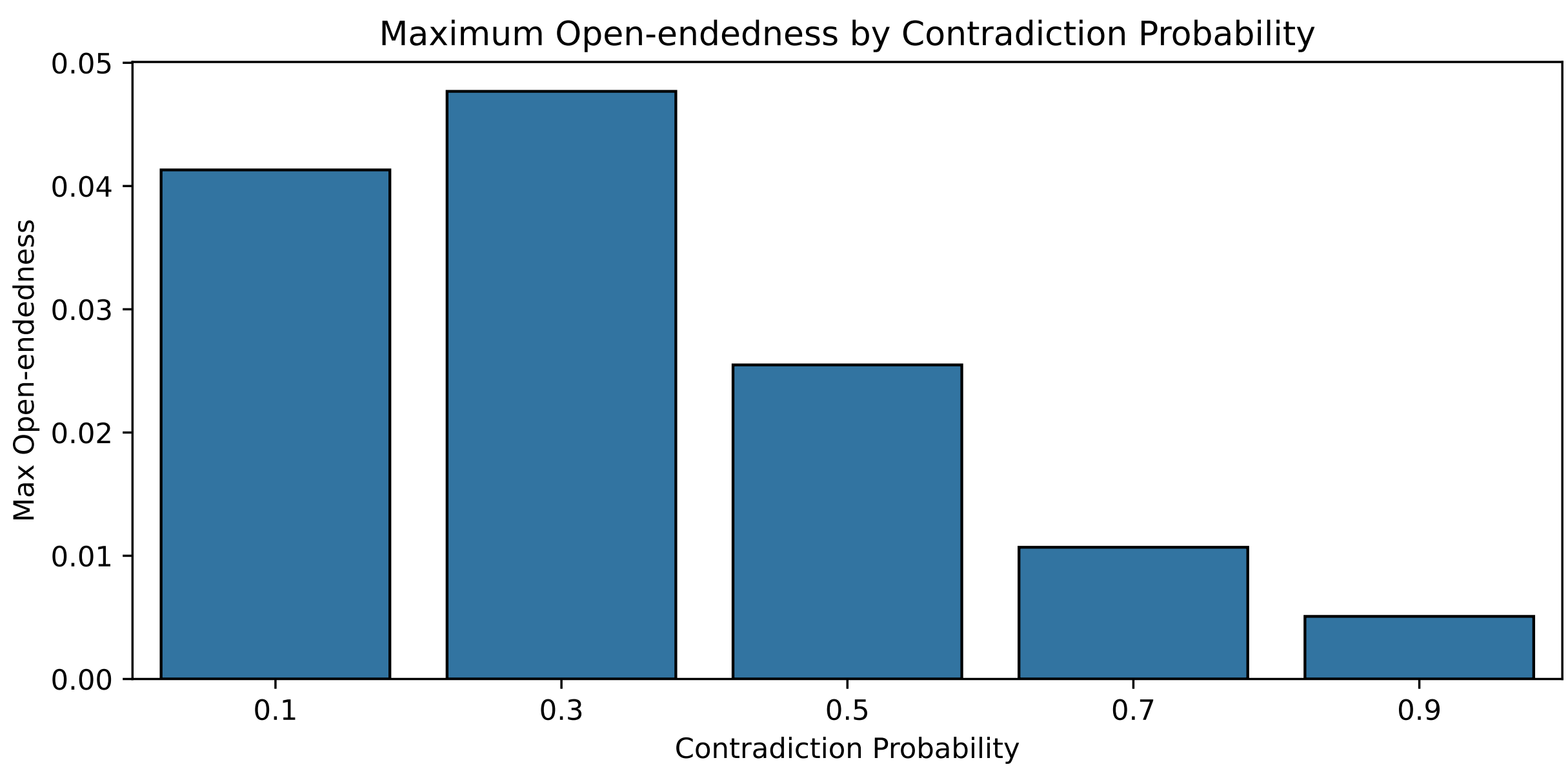

Supplementary Figure 17: **Peak Ω across K at each c.** Highlights how c balances exploration (more cycles found) against stability (dwell times).

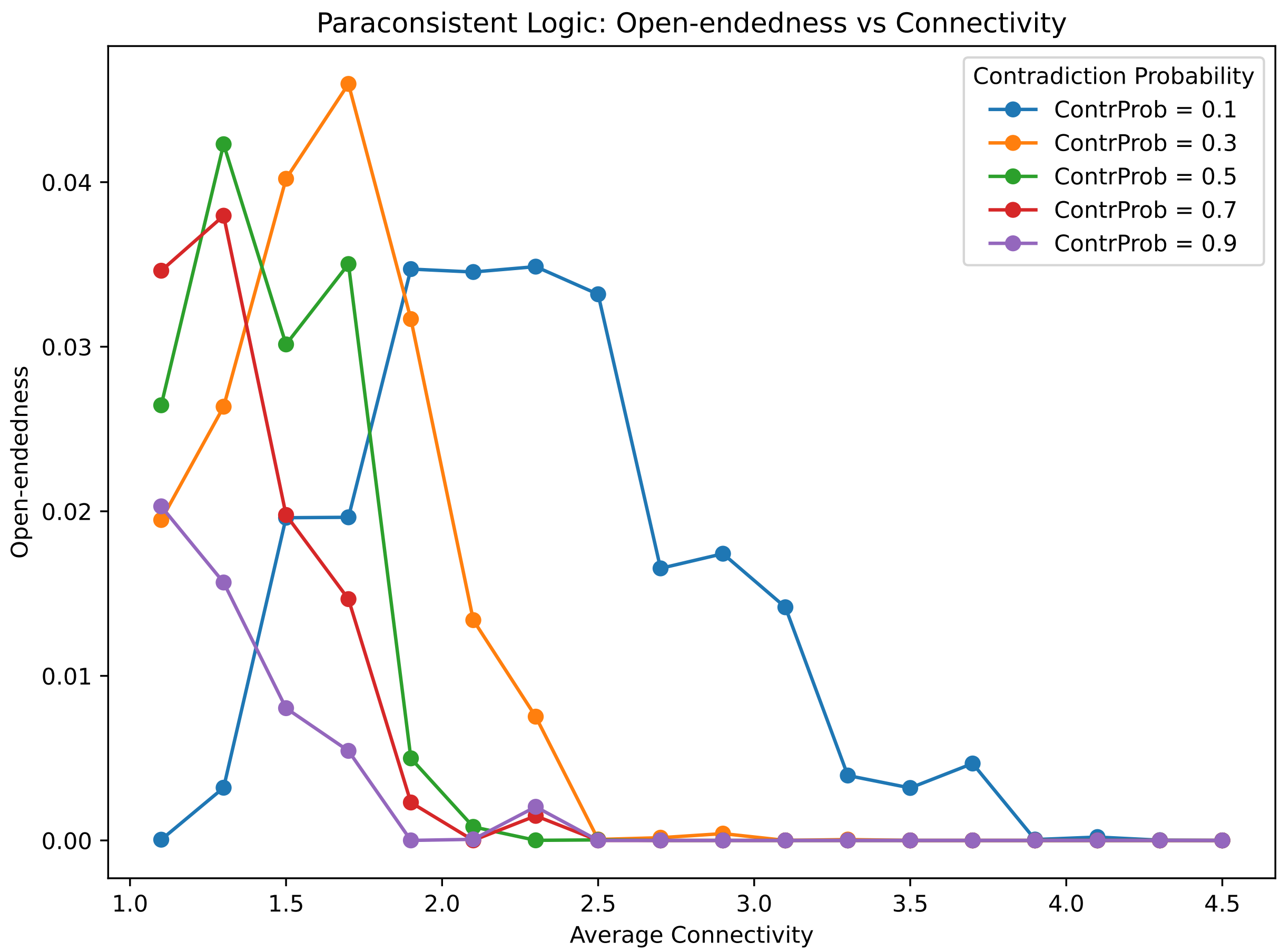

Supplementary Figure 18: $\Omega(K)$ **with heterogeneity under paraconsistency.** Broad shoulder near $K \in [1.5, 2.3]$ reproduces the criticality-extending effect of heterogeneity.

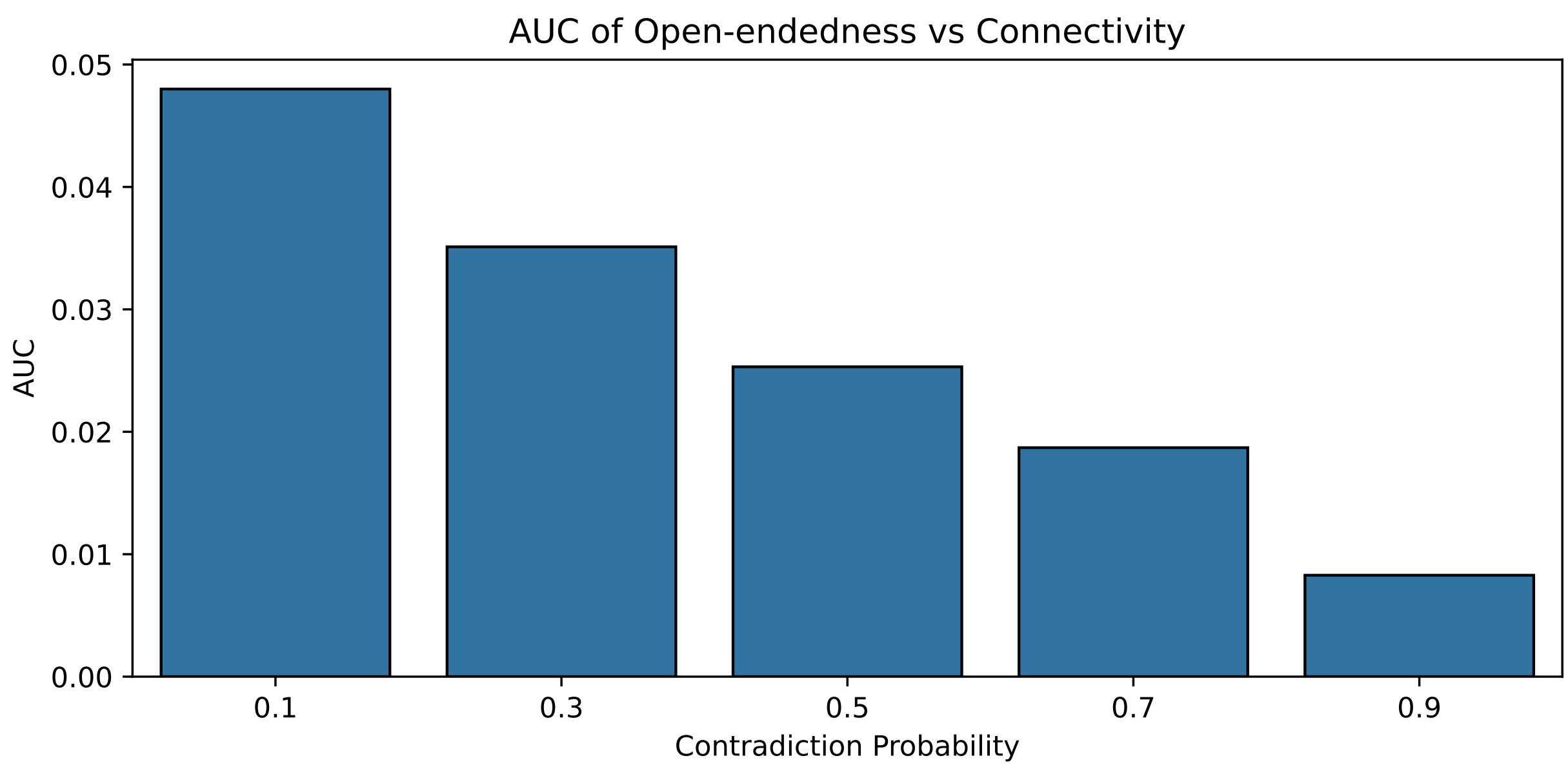

Supplementary Figure 19: **AUC vs. c under heterogeneity.** Confirms the preference for small but non-zero c (best: `ContrProb=0.1`).

Modal logic: accessibility degree a, possibility p_{poss}, necessity p_{necc}

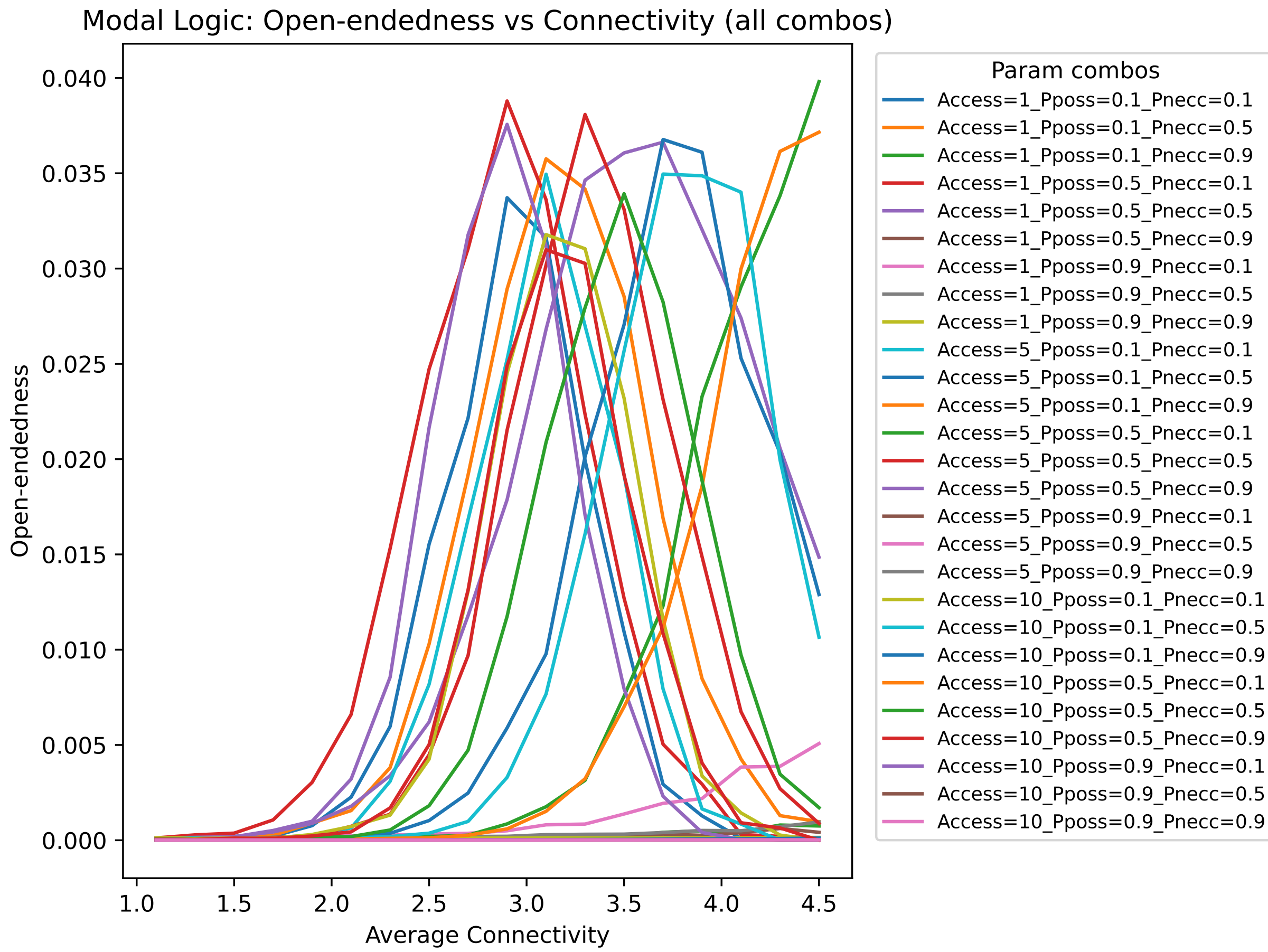

Supplementary Figure 20: **All $\Omega(K)$ curves across $(a, p_{\text{poss}}, p_{\text{necc}})$ combinations.** Necessary gates act as canalizing constraints; possible gates reopen paths contextually. The best combination is `Access=1_Pposs=0.5_Pnecc=0.5`, as shown in Table 1.

Supplementary Table 1: **Area under the curve, maximum OE and associated connectivity for all combinations of modal parameters (Homogeneous Case)**. Values are ranked from highest to lowest AUC.

	Access	Pposs	Pnecc	AUC	MaxOE	ConnAtMax
0	1	0.5	0.5	0.052871	0.036631	3.7
1	1	0.5	0.1	0.039701	0.038794	2.9
2	1	0.1	0.5	0.039010	0.035759	3.1
3	5	0.1	0.5	0.038415	0.036771	3.7
4	5	0.5	0.5	0.037514	0.038086	3.3
5	10	0.1	0.5	0.036748	0.034959	3.7
6	5	0.5	0.9	0.032701	0.037566	2.9
7	10	0.5	0.5	0.032658	0.033927	3.5
8	1	0.1	0.1	0.029547	0.033719	2.9
9	10	0.1	0.1	0.029440	0.031780	3.1
10	5	0.1	0.1	0.029239	0.034953	3.1
11	10	0.5	0.9	0.028516	0.030986	3.1
12	5	0.5	0.1	0.026492	0.039809	4.5
13	10	0.5	0.1	0.025501	0.037157	4.5
14	1	0.9	0.1	0.003950	0.005074	4.5
15	1	0.9	0.5	0.001013	0.000958	4.5
16	1	0.1	0.9	0.000693	0.000798	4.3
17	1	0.5	0.9	0.000636	0.000617	4.3
18	1	0.9	0.9	0.000423	0.000148	4.5
19	10	0.1	0.9	0.000137	0.000118	4.5
20	5	0.1	0.9	0.000081	0.000040	4.3
21	10	0.9	0.9	0.000057	0.000021	4.3
22	10	0.9	0.5	0.000048	0.000017	4.3
23	10	0.9	0.1	0.000043	0.000015	4.1
24	5	0.9	0.5	0.000042	0.000015	4.5
25	5	0.9	0.9	0.000042	0.000013	3.7
26	5	0.9	0.1	0.000042	0.000014	4.3

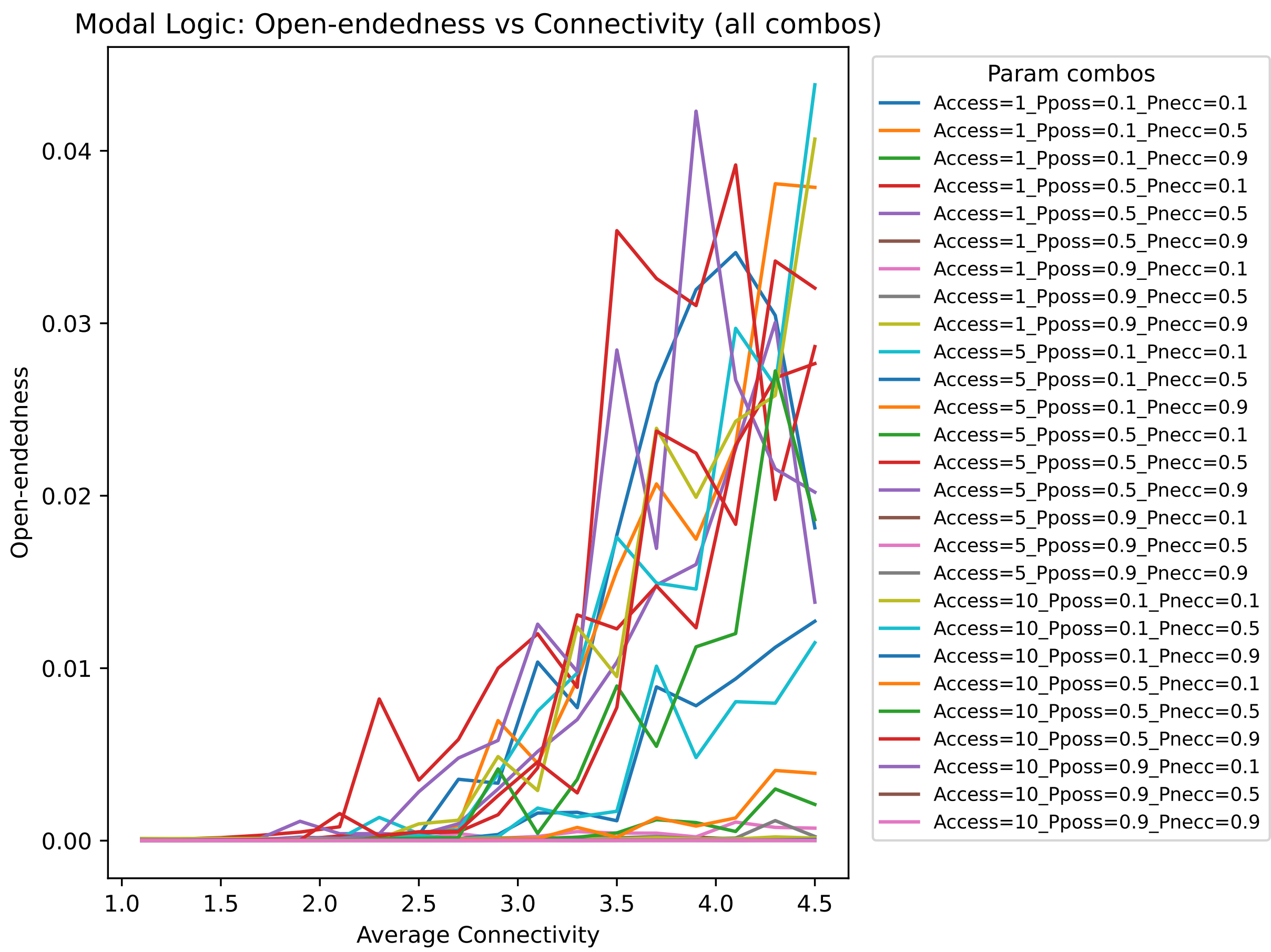

Supplementary Figure 21: $\Omega(K)$ **across** $(a, p_{\text{poss}}, p_{\text{necc}})$ **under heterogeneity.** The winning combo used in the main text is `Access=1_Pposs=0.5_Pnecc=0.1`, which preserves metastable corridors at high K.

Supplementary Table 2: **Area under the curve, maximum OE and associated connectivity for all combinations of modal parameters (Heterogeneous Case)**. Values are ranked from highest to lowest AUC.

	Access	Pposs	Pnecc	AUC	MaxOE	ConnAtMax
0	1	0.5	0.5	0.052871	0.036631	3.7
1	1	0.5	0.1	0.039701	0.038794	2.9
2	1	0.1	0.5	0.039010	0.035759	3.1
3	5	0.1	0.5	0.038415	0.036771	3.7
4	5	0.5	0.5	0.037514	0.038086	3.3
5	10	0.1	0.5	0.036748	0.034959	3.7
6	5	0.5	0.9	0.032701	0.037566	2.9
7	10	0.5	0.5	0.032658	0.033927	3.5
8	1	0.1	0.1	0.029547	0.033719	2.9
9	10	0.1	0.1	0.029440	0.031780	3.1
10	5	0.1	0.1	0.029239	0.034953	3.1
11	10	0.5	0.9	0.028516	0.030986	3.1
12	5	0.5	0.1	0.026492	0.039809	4.5
13	10	0.5	0.1	0.025501	0.037157	4.5
14	1	0.9	0.1	0.003950	0.005074	4.5
15	1	0.9	0.5	0.001013	0.000958	4.5
16	1	0.1	0.9	0.000693	0.000798	4.3
17	1	0.5	0.9	0.000636	0.000617	4.3
18	1	0.9	0.9	0.000423	0.000148	4.5
19	10	0.1	0.9	0.000137	0.000118	4.5
20	5	0.1	0.9	0.000081	0.000040	4.3
21	10	0.9	0.9	0.000057	0.000021	4.3
22	10	0.9	0.5	0.000048	0.000017	4.3
23	10	0.9	0.1	0.000043	0.000015	4.1
24	5	0.9	0.5	0.000042	0.000015	4.5
25	5	0.9	0.9	0.000042	0.000013	3.7
26	5	0.9	0.1	0.000042	0.000014	4.3

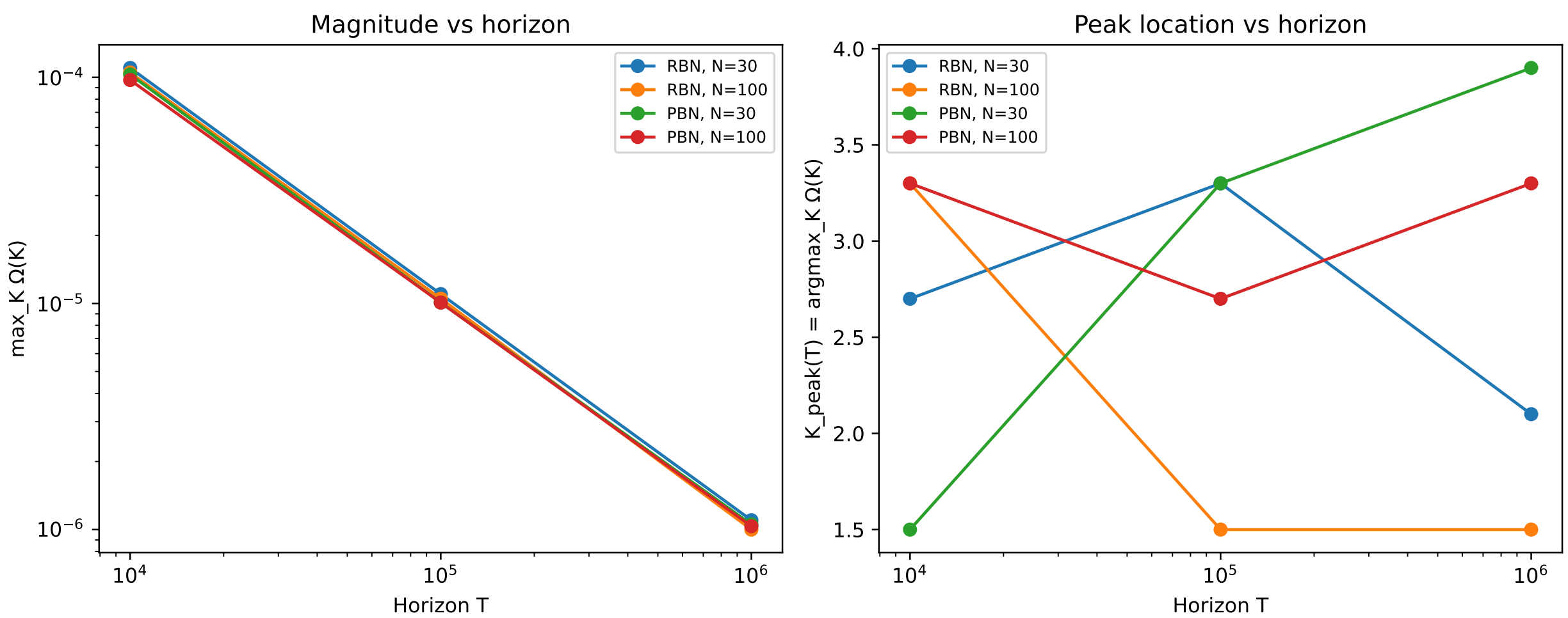

Supplementary Figure 22: $\Omega(T)$ **convergence across model classes (deterministic RBN vs stochastic PBN) with small-N control.** For each horizon $T \in \{10^4, 10^5, 10^6\}$ we computed $\Omega(K)$ on a coarse K grid and summarized (left) the peak magnitude $\max_K \Omega(K)$ and (right) the peak location $K_{\mathrm{peak}}(T) = \arg\max_K \Omega(K)$. Across both the deterministic baseline (RBN) and a stochastic switching mechanism (PBN), $\max_K \Omega(K)$ decays approximately as $1/T$ over the tested horizons, confirming the finite-horizon nature of Ω and explaining the observed tendency toward zero at long T. In contrast, $K_{\mathrm{peak}}(T)$ can move with T because increasing the observation window changes the balance between transient exploration and time spent in the recurrent structure. The right panel of this figure shows that this finite-horizon peak location is mechanism- and size-dependent. Because $K_{\mathrm{peak}}(T)$ is computed on a coarse K grid (5 points), it should be interpreted qualitatively (direction/shifts), not as a high-resolution estimate of the optimal K.

Abbreviations

ARM: Annealed Rule Mutation; PBN: Probabilistic Boolean Network; K: average in-degree; T: number of time steps; AUC: area under the $\Omega(K)$ curve.

Notes on computation and data flow

- **Metric extraction.** The notebooks rely on the extractor that computes (V, P, KD) and $\Omega = KD/T^2$ from streams of states, ensuring correctness for both CPU and GPU paths.
- **GPU/CPU paths.** Long runs stream trajectories on the GPU when available; modest horizons use CPU multiprocessing. The cycle–tail shortcut is used for very large T when appropriate to avoid unnecessary simulation.
- **AUC definition.** Unless otherwise indicated, AUC is computed by trapezoidal integration of the mean $\Omega(K)$ curve over the K grid.

External benchmarks, baseline indices, and symmetry control

Elementary Cellular Automata (ECA)

To provide an external "known dynamical systems" validation, we evaluated Ω on a small panel of classic Wolfram ECA rules $\{0, 4, 30, 54, 90, 110, 150, 184, 255\}$ with periodic boundary conditions and random initial states, using $N = 100$ cells and horizon $T = 200,000$. Fixed-point rules (e.g., 0 and 255) rapidly settle and yield $\Omega(T) \approx 1/T$, while other rules exhibit larger Ω due to longer-lived recurrent structure. Notably, some rules can yield $\Omega \approx 0$ within a finite horizon if exact microstate recurrences are not observed, illustrating that Ω tracks recurrence-weighted persistence rather than raw novelty.

As shown in Figure S23, among the tested rules, Rule 54 attains the largest mean Ω. This is plausible because Rule 54 is a canonical Class-IV automaton: on finite periodic lattices it supports moving localized structures whose interactions can sustain long-lived recurrent episodes, exactly the type of behavior that Ω rewards. By contrast, Rules 90 and 150 also yield comparatively large Ω values, even though they are not Class-IV in the usual Wolfram sense, because they are additive rules. On finite rings, their linear dynamics over GF(2) generate exact recurrences and often long global periods, which a recurrence-based metric captures efficiently. Rule 110, although also Class-IV, yields a smaller Ω under our protocol because random initial conditions on a size-100 periodic lattice do not necessarily produce exact whole-lattice recurrences as efficiently within the finite observation window. A fuller account of how Wolfram class, additivity, lattice size, and initial-condition ensemble jointly shape Ω lies beyond the scope of the present paper, but these comparisons already show that Ω is sensitive to finite-horizon recurrence structure rather than to Wolfram class labels alone.

To address any symmetry concern, we recomputed Ω under the complement quotient that identifies each Boolean state s with its complement $1 - s$ by canonicalizing each visited state to $\min(s, 1 - s)$. In this way, the qualitative ordering is unchanged under this quotient.

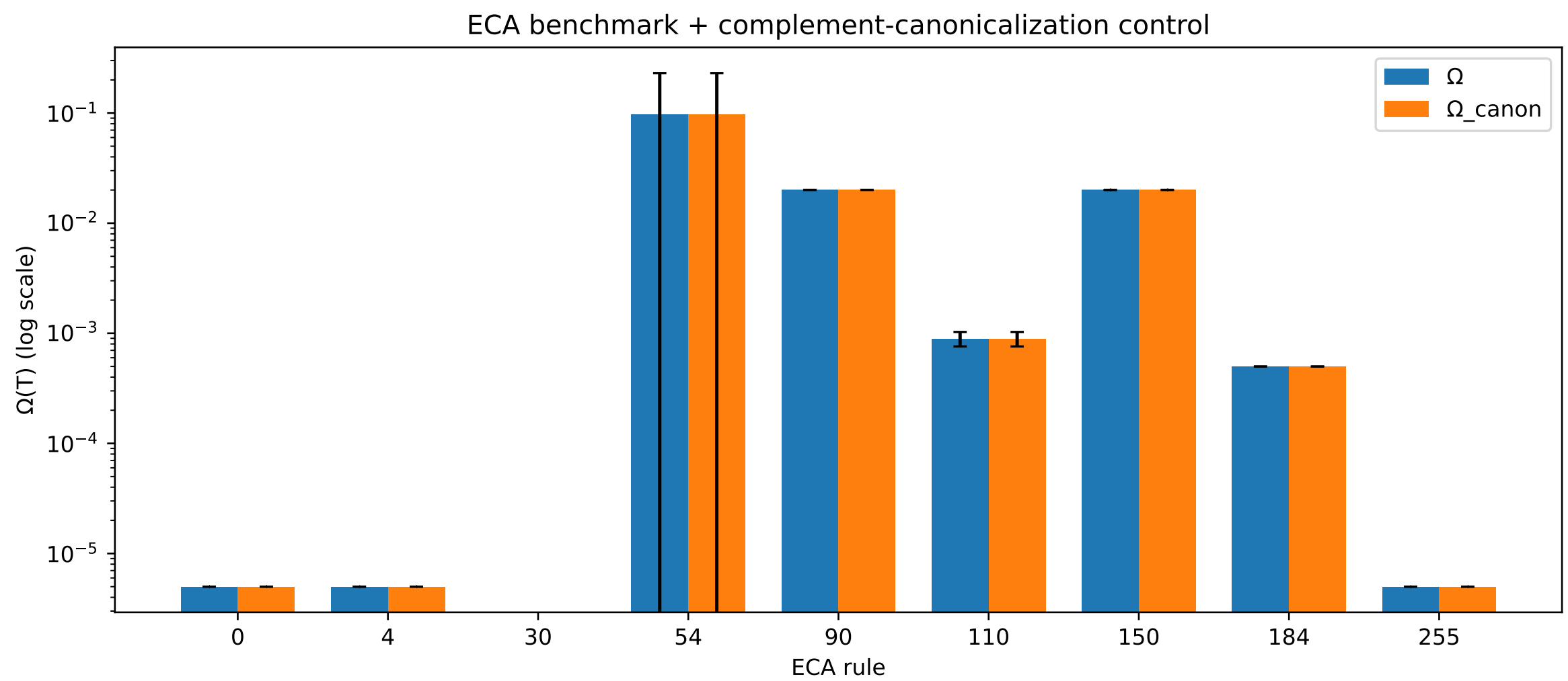

Supplementary Figure 23: **ECA benchmark and complement-symmetry control.** Mean Ω across seeds for a panel of classic ECA rules. Rule 54 attains the largest average value in this protocol, while additive Rules 90 and 150 also score highly because finite-ring linear dynamics generate exact recurrences. We also report Ω_{canon} computed after canonicalizing each state under the complement quotient $s \sim 1 - s$ (mapping to $\min(s, 1 - s)$).

Comparing Ω to baseline novelty/complexity proxies

To clarify what Ω measures relative to standard trajectory-level summaries, we computed three baselines on the same runs: (i) unique-state fraction $|\{x(t)\}|/T$, (ii) mean node entropy, and (iii) a simple compression proxy (zlib-compressed size divided by raw size). Figure S24 shows that Ω is not reducible to these baselines: high novelty/noise (high unique-state fraction or entropy) does not necessarily produce high Ω if recurrence is weak, while structured recurrence can yield higher Ω even when novelty proxies are moderate.

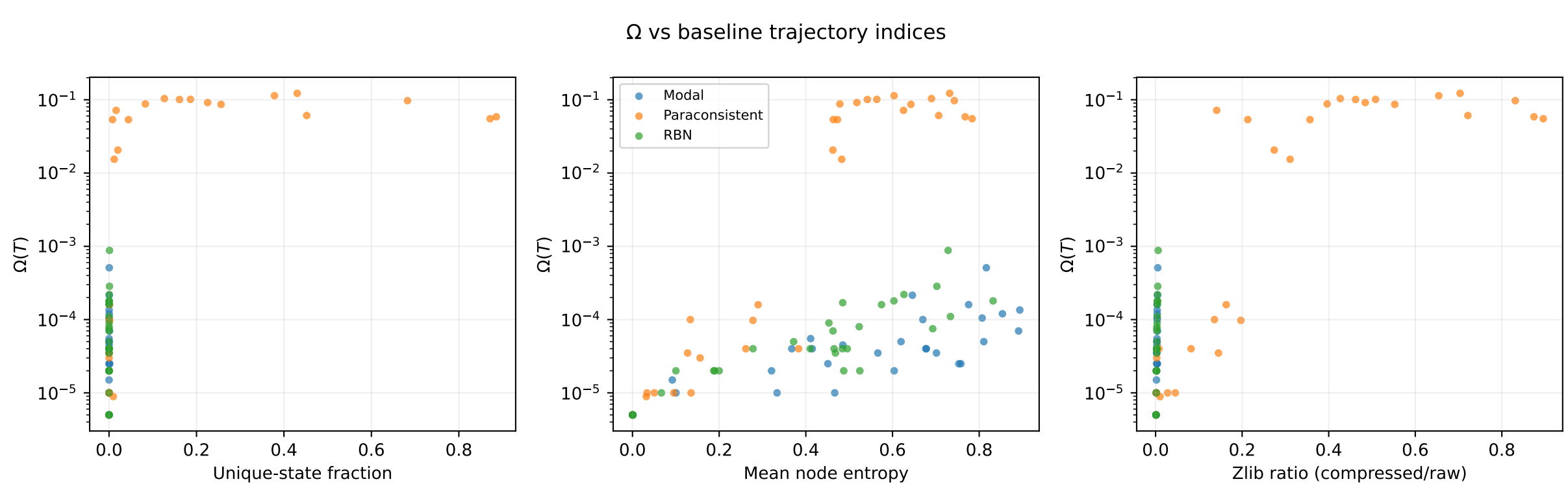

Supplementary Figure 24: **Baseline index comparisons.** Scatter plots of Ω versus (i) unique-state fraction, (ii) mean node entropy, and (iii) a compression proxy. These comparisons support the interpretation that Ω tracks recurrence-weighted persistence rather than undifferentiated variability.

Curated Boolean GRN models

We next computed Ω on curated Boolean GRN models (Cell Collective models exported to BoolNet `.bnet` format). Across all tested models and seeds, the curated GRNs remain clustered near

$\Omega(T) \approx 1/T$, consistent with rapid settling to fixed points or short cycles (i.e., stable cell-fate attractors) rather than sustained novelty. This supports the interpretation that many single-circuit GRN models are biologically designed to be homeostatic and therefore are not expected to be open-ended; OEE-like behavior is more naturally sought at population, ecology-of-networks, or evolving-circuit scales.

To make the scale of these values easier to interpret, Figure S25 also includes size-matched classical RBN reference ensembles at three representative connectivities ($K = 1.5, 2.1, 3.5$), constructed with Poisson in-degree, synchronous updating, and fixed bias 0.5, using the median curated-model size ($N = 22$). These reference ensembles are included only as scale controls: in small deterministic RBNs, $\Omega(T)$ is expected to behave approximately as λ/T once the eventual cycle is reached, so the benchmark should not be read as a statement that one connectivity regime is intrinsically "more open-ended" than another. Instead, the purpose of the comparison is simply to show that curated GRNs lie close to the finite-horizon floor, whereas generic classical Boolean controls of similar size can realize broader recurrence scales. Complement-canonicalization changed the corresponding values only negligibly and is omitted from the figure for readability.

Implementation note for reproducibility: any regulator that appeared only on the RHS of an update rule (never as an LHS defined variable) was treated as an exogenous input with identity update (frozen value), initialized randomly per seed. The model selection and model curation were based on the detailed analysis carried out by Park, K. H., Costa, F. X., Rocha, L. M., Albert, R., & Rozum, J. C. (2023). *Models of cell processes are far from the edge of chaos.* PRX Life, 1(2), 023009.

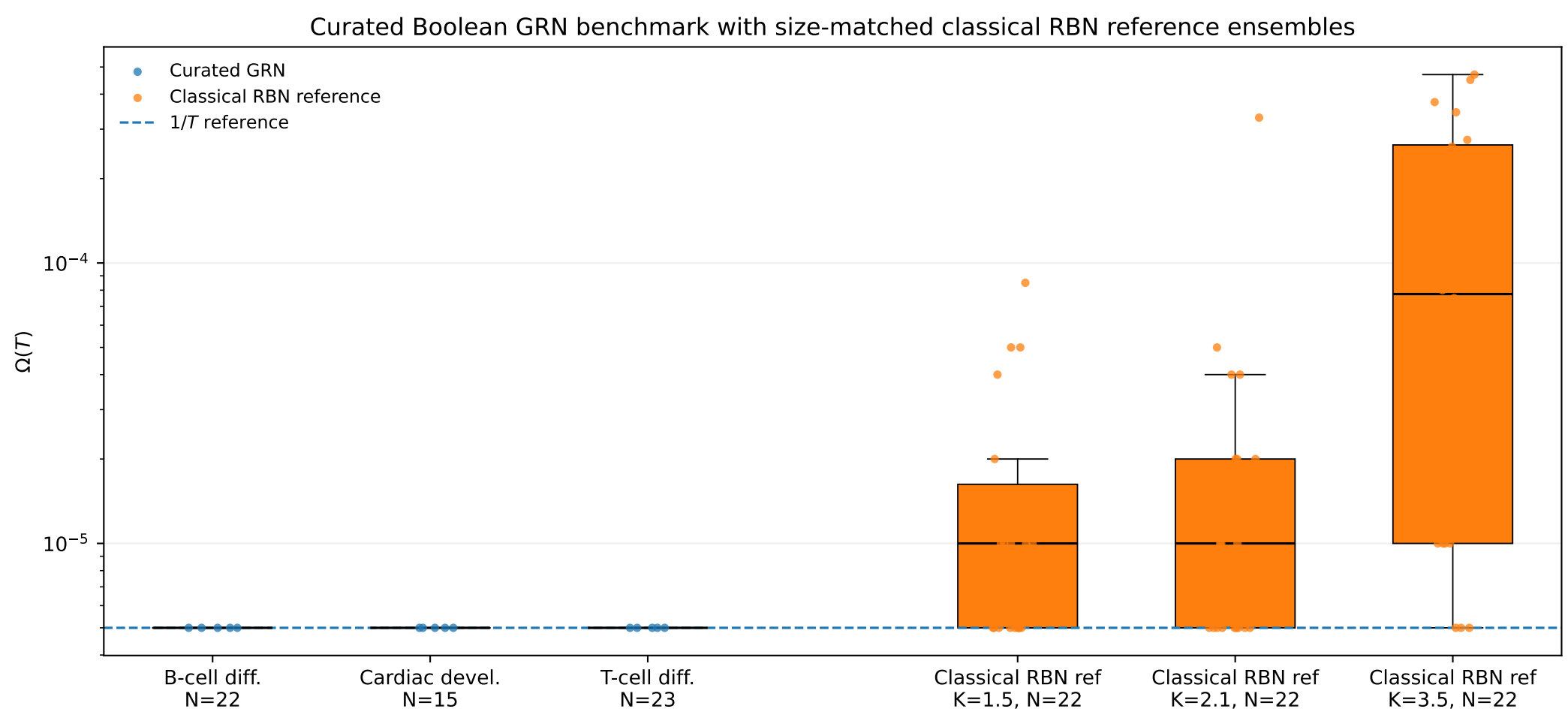

Supplementary Figure 25: **Curated Boolean GRN benchmark with size-matched classical RBN reference ensembles.** Distributions of Ω computed on curated Boolean GRN models across seeds/initial conditions, together with three size-matched classical RBN reference ensembles at representative connectivities ($K = 1.5, 2.1, 3.5$). The dashed line marks the $1/T$ baseline. The curated GRNs remain clustered near this scale, consistent with stable regulatory dynamics, while the classical RBN controls span a broader recurrence range. These reference ensembles are included as scale controls rather than as a phase-classification test.

Interpretive scope of the benchmark analyses. The ECA and curated-GRN benchmarks are intended as controlled tests of finite-horizon recurrence structure, not as complete empirical validation of biological OEE. In all cases, $\Omega(T)$ is conditional on the chosen state representation, update

scheme, observation window, and coarse-graining. In particular, hidden variables or unobserved regulatory degrees of freedom could either create apparent recurrences in a projected state space or obscure recurrence that exists in the full system. The curated GRN results should therefore be interpreted as showing that these fixed Boolean regulatory models are close to the recurrence floor under the tested conditions, consistent with homeostatic or cell-fate-stabilizing dynamics, rather than as a claim that GRNs as evolving populations or ecological ensembles cannot participate in OEE-like processes.
